# Antimicrobial activities of metabolites isolated from endophytic *Aspergillus* *flavus* of *Sarcophyton* *ehrenbergi* supported by in-silico study and NMR spectroscopy

**DOI:** 10.1186/s40694-023-00161-2

**Published:** 2023-08-02

**Authors:** Abdel Nasser B. Singab, Yasmin A. Elkhawas, Eman Al-Sayed, Ahmed M. Elissawy, Iten M. Fawzy, Nada M. Mostafa

**Affiliations:** 1 Department of Pharmacognosy, Faculty of Pharmacy, Ain-Shams University, Cairo, 11566 Egypt; 2 Center of Drug Discovery Research and Development, Ain-Shams University, Cairo, 11566 Egypt; 3 Department of Pharmacognosy and Medicinal Plants, Faculty of Pharmacy, Future University in Egypt, Cairo, 11835 Egypt; 4 Department of Pharmaceutical Chemistry, Faculty of Pharmacy, Future University in Egypt, Cairo, 11835 Egypt

**Keywords:** In silico profile, Antimicrobial, Anticancer, Endophyte, Dynamic simulation, NMR spectroscopy, *Sarcophyton ehrenbergi*

## Abstract

**Background:**

Endophytic *Aspergillus* species produce countless valuable bioactive secondary metabolites. In the current study, *Aspergillus flavus* an endophyte from the soft coral *Sarcophyton ehrenbergi* was chemically explored and the extracted phytoconstituents were subsequently evaluated for antimicrobial activity. This is accomplished by employing nuclear magnetic resonance (NMR) spectroscopy and computational techniques. Additionally, An in vitro anticancer analysis of *A. flavus* total extract against breast cancer cells (MCF-7) was investigated.

**Result:**

Six compounds were separated from the crude alcohol extract of the endophytic *Aspergillus flavus* out of which anhydro-mevalonolactone was reported for the first time. The anti-fungal and anti-*Helicobacter pylori* properties of two distinct compounds (Scopularides A and B) were assessed. Additionally, computational research was done to identify the binding mechanisms for all compounds. Both the compounds were found to be active against *H. pylori* with minimum inhibitory concentration (MIC) values ranging from 7.81 to 15.63 µg/ mL as compared with clarithromycin 1.95 µg/ mL. Scopularides A was potent against both *Candida albicans* and *Aspergillus niger* with MIC values ranging from 3.9 to 31.25 µg/ mL, while scopularides B only inhibits *Candida albicans* with MIC value of 15.63 µg/ mL and weak inhibitory activity against *A. niger* (MIC = 125 µg/ mL). Furthermore, cytotoxic activity showed a significant effect (IC_50_: 30.46 mg/mL) against MCF-7 cells.

**Conclusion:**

Our findings report that cytotoxic activity and molecular docking support the antimicrobial activity of *Aspergillus flavus*, which could be a promising alternative source as a potential antimicrobial agent.

**Supplementary Information:**

The online version contains supplementary material available at 10.1186/s40694-023-00161-2.

## Background

In the twenty-first century, severe infections caused by bacteria, resistant to frequently used antibiotics have become a major global healthcare concern [1]. Antimicrobial resistance has also required more time and effort to treat, making the issue both more serious and time-consuming [2]. Microbial resistance to antibiotics is linked to changes in antibiotic permeability, target molecules, enzymatic degradation of medications, and intrinsic antimicrobial efflux from the cytosol. These strategic tactics are used by bacteria and other microbes to prevent antibiotic toxicity [3]. The production of new antibiotics has dramatically decreased in recent years. Instead, existing drugs are being reviewed more often, and efforts are being made to develop novel antibiotics [3]. Traditional medicinal herbs, used from ancient times [4] are expected to be the natural source of more potential antibiotics in the future. Several ways have been tried to discover novel antimicrobial chemicals or to increase the activity of current antibacterial medications which are scarcely derived from endophytic fungi of medicinal plants [5]. The quest for new bioactive metabolites from marine resources and their isolated endophytes is rising today because of their unique metabolites [6–8]. *Helicobacter ppylori* is the most common cause of chronic gastritis, peptic ulcers, and gastric cancer. It continues to be a serious infectious disease that affects adults in underdeveloped nations. Due to the emergence of multidrug-resistant strains during the past few years, existing control efforts have mostly been limited. This has shifted dramatically the focus of the scientific community towards harnessing natural resources, particularly plants and fungi to develop safer and more valuable drugs [9].

Fungal infections can be hazardous for patients, particularly for those using anticancer or immunosuppressive medications. The patient is more vulnerable to bacterial, viral, and fungal infections due to immunosuppression imposed on illnesses or other disorders. Due to the damage caused by fungal infections while using immunosuppressive medications, such as anticancer medications, safer antifungal medications have been developed with minimal negative clinical effects. Since many of the present medications had adverse effects, losing their effectiveness against new fungal strains, or the intended fungi had grown resistant to them, the demand for the next-generation antifungal agents has expanded. Therefore, it is crucial to develop antifungal medications with a broad spectrum of biocidal activity, low toxicity, and a novel mode of action. Most of the anti-infective medications in the industry were produced from the vast diversity of bioresources, which can act as an inexhaustible reservoir of naturally occurring bioactive secondary chemicals [10, 11]. Natural products offer effective alternatives and promising sources for many bioactivities [12, 13].

Infections triggered by drug-resistant microorganisms are becoming more common all over the world. Furthermore, many adverse effects linked to the consistent use of antifungal medicines have been recorded. Systemic fungal infections have been treated using amphotericin B, which is produced from *Streptomyces nodosus*. However, it was well known to have dangerous, perhaps fatal adverse effects like high temperature and trembling chills after a few hours of infusion. Azole antifungal medications are commonly used in treating a wide range of fungal illnesses. Their antifungal strategies include decreasing the formation of membrane ergosterol, which inhibits fungal growth via CYP450 suppression. However, these medicines have e potential to adhere to human membranes and cause kidney injury [14]. As a result, new medications must be discovered from a variety of sources. Many isolated natural chemical compounds and extracts have been reported for their potential biological activities [15–17]. Natural sources such as terrestrial and marine endophytes and their bioactive metabolites are being investigated for their antimicrobial activity and due to their distinctiveness, the search for novel bioactive metabolites from marine natural resources and their derived endophytes is continuously increasing [6–8]. Fungistatic and fungicidal properties can be found in many natural products or components. These pharmaceutically valuable agents of natural origins have significantly lesser side effects than synthetic ones [9].

Molecular docking is a simulation technique which determines the optimal fit between ligands and the active sites of certain targets. This process entails assembling a 3D coordinate space on the target's binding site, calculating the binding affinity of the generated complex, and elaborating the molecule's subsequent orientation on the ligand's binding site [18]. The biggest negative number represents the sensitivity of binding affinity, which is a representation of the best shape or created complex [19, 20].

Since endophytes, abundant in tissues of almost all marine and terrestrial plants so far, have been acknowledged as a valuable source of bioactive secondary metabolites, we evaluated the antimicrobial activity of cyclodepsipeptides against *Helicobacter pylori, Candida albicans,* and *Aspergillus niger*. According to previously reported data, polyketides and cyclodepsipeptide showed antimicrobial activity [21], which prompted us to comprehensively evaluate the antimicrobial activities of isolated metabolites, together with a prediction of the antimicrobial activity of the isolated compounds by using molecular docking.

## Results

### Identification of fungal crude extract separated compounds

**Fig. 1:**
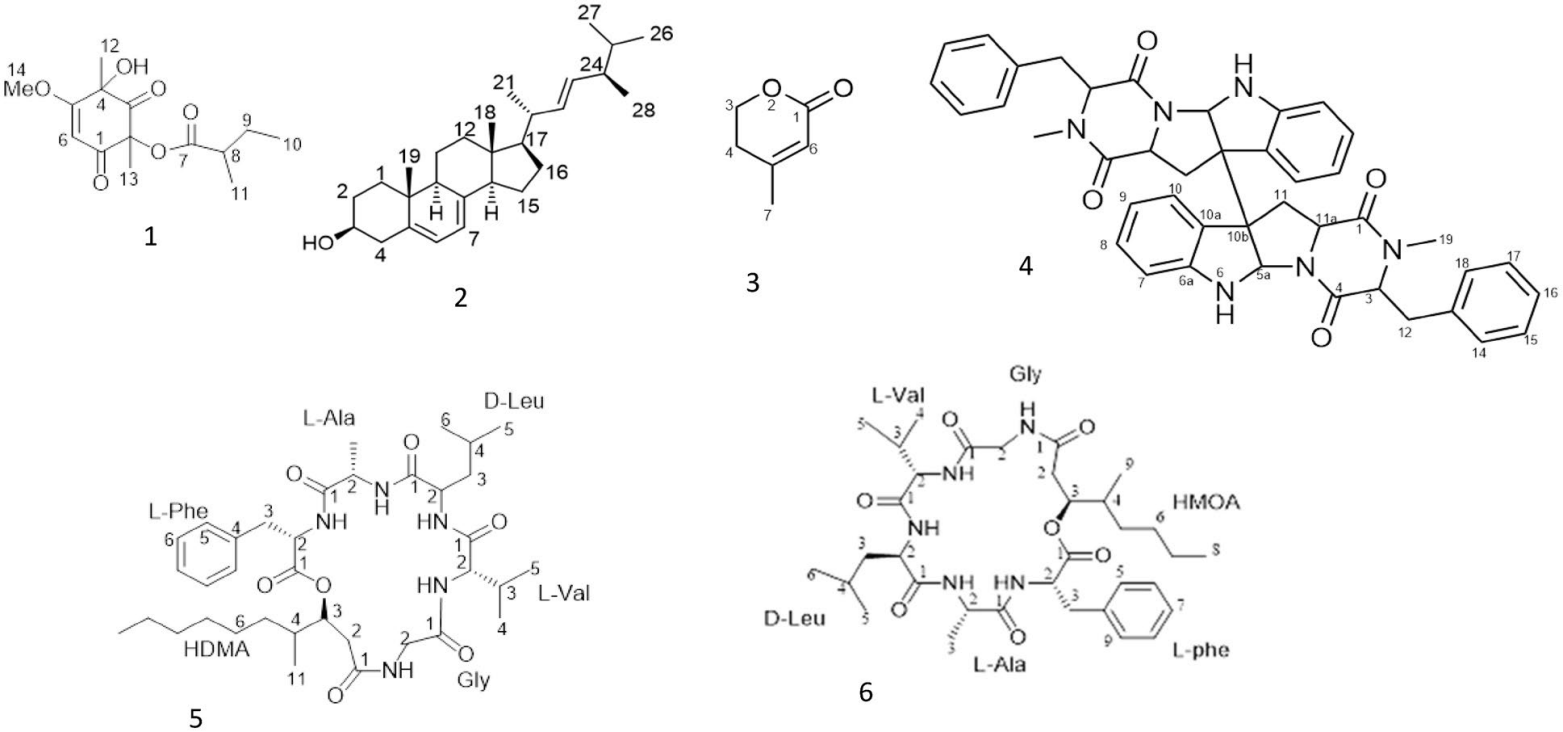
Structures of the isolated compounds from *Aspergillus flavus*; Phomaligol A (1), Ergosterol (2), Anhydro-mevalonolactone (3), Ditryptophenaline (4), scopularide A (5) and scopularide B (6)

To investigate the active metabolites, present in the identified fungus, the purification of crude extracts was performed, and this yielded six main compounds, namely, phomaligol A (1), Ergosterol (2), anhydro-mevalonolactone (3), Ditryptophenaline (4), and scopularide A (5) and B (6) (Fig. 1) The separated compounds were identified based on their spectral data (^1^H-, ^13^C-NMR), and were compared with the compounds already reported in the literature.

Phomaligol A was separated as a colorless oil, and NMR data (CDCl_3,_ 25 ºC) were in agreement with those reported for phomaligol A (Additional file 1: Figs. S1, S2) [22]. Ergosterol was separated as colorless crystals, which was confirmed by reported data on ergosterol (Additional file 1: Figs. S3, S4) [23, 24].

Anhydro-mevalonolactone was separated as a pale-yellow oil. The ^1^H-NMR [(DMSO_,_ 25 ºC)] spectral analysis showed the presence of two protons at ^δ^H 2.39 (t, 2H, H-4) and 4.40 (t, 1H, H-3), methyl protons as a singlet at ^δ^H 1.96 (3H, H-7) and a methine proton at ^δ^H 5.74 (m, 1H, H-6). The structural assignment of compound 3 was further substantiated by the attached proton test (APT) experiment. It presents six-member lactone ring with a carboxylic carbon at ^δ^C 164.39 (C-1), ethylene carbon at 115.94 (C-6) and three aliphatic carbons at ^δ^C 66.1 (C-3), ^δ^C 29.09 (C-4) and ^δ^C 22.9 (C-7) ppm, and one quaternary carbon at ^δ^C 160.64 (C-5). For compound elucidation using HMBC, the carbon at 160.64 (C-5) linked with the methyl group at ^δ^H 1.96 (H-7) showed a long range coupling with ^δ^H 2.38 (H-4) and ^δ^H 4.40 (H-3). The carbonyl carbon showed long-range coupling with (H-3). Moreover, C-4 showed a relation with a methyl group at ^δ^H 1.96 ppm (H-7) and another coupling with methylene proton at ^δ^H 4.40 (H-3) ppm. The ^1^H-^1^H COSY of compound 3, showed a correlation between H-3 at ^δ^H 4.40 (t, J = 6.3 Hz) and H-4 at ^δ^H 2.38 (t, J = 6.4 Hz). This contribution represents the first report on the isolation and identification of this compound from *A. flavus*. The NMR data were in accordance with those reported for the fungal metabolite anhydro-mevalonolactone in the literature (Additional file 1: Fig. S5–S8) [25]. Ditryptophenaline is a white solid and the NMR data (CDCl_3,_ 25 ºC) were in accordance with those reported for the fungal metabolite ditryptophenaline (Additional file 1: Fig. S9–S12) [26, 27]. Scopularide A and B (NMR data recore in CDCl_3,_ 25 ºC) were isolated as previously reported in [19] (Additional file 1: Fig. S13–S19).

### Antimicrobial activity of the separated cyclodepsipeptides

To achieve the aim of the study, both cyclodepsipeptides were investigated for their antifungal and anti-*H*. *Pylori* activities. Scopularide A showed a potent broad-spectrum antifungal activity against both *Candida albicans* and *A*. *niger* with MIC values ranging from 3.9 to 31.25 µg/ ml, while Scopularide B with a MIC value 15.63 µg/ml showed potent activity against *Candida albicans*, and minimal activity against *A*. *niger* (MIC = 125 µg/ ml) (Table 1).

**Table 1:** Mean inhibitory percentages of Scopularide (A and B) and Amphotericin B against *Candida albicans* and *Aspergillus niger*. and Clarithromycin against *H. pylori.* All tests were carried out in triplicate and values are expressed as mean ± SD

Sample concentration (µg/ml)	Mean inhibitory percentages against *Candida albicans*		
	Amphotericin B	Scopularide A	Scopularide B
125	100	100	100
62.5	100	100	100
31.2	100	100	100
15.6	100	100	100
7.8	100	100	88.6 ± 1.9
3.9	100	100	65.1 ± 2.1
1.9	100	81.3 ± 1.3	49.3 ± 1.5
0.9	100	59.3 ± 0.6	28.1 ± 0.5
0.4	79.3 ± 1.3	34.2 ± 2.2	11.0 ± 2.1
0.2	54.3 ± 2.5	16.8 ± 1.7	4.9 ± 1.3
0	0	0	0
MIC	0.9	3.9	15.6

Sample concentration (µg/ml)	Mean inhibitory percentages against *Aspergillus niger*		
	Amphotericin B	Scopularide A	Scopularide B
125	100	100	100
62.5	100	100	82.1 ± 1.7
31.2	100	100	63.1 ± 2.2
15.6	100	86.3 ± 1.3	48.3 ± 1.5
7.8	100	71.2 ± 0.58	19.3 ± 2.1
3.9	100	56.3 ± 2.1	7.4 ± 1.6
1.9	83.2 ± 1.6	38.2 ± 1.5	0
0.9	69.2 ± 0.8	21.0 ± 0.6	0
0.4	54.1 ± 1.4	8.3 ± 1.2	0
0.2	39.2 ± 0.5	0	0
0	0	0	0
MIC	3.9	31.2	125

Sample concentration (µg/ml)	Mean inhibitory percentages against *H. pylori*		
	Clarithromycin	Scopularide A	Scopularide B
125	100	100	100
62.5	100	100	100
31.2	100	100	100
15.6	100	100	100
7.8	100	100	86.3 ± 1.3
3.9	100	92.4 ± 1.5	76.8 ± 2.1
1.9	100	84.7 ± 0.5	62.1 ± 0.9
0.9	92.4 ± 1.2	76.2 ± 0.5	52.1 ± 1.6
0.4	87.6 ± 0.58	54.1 ± 1.6	42.3 ± 2.2
0.2	81.3 ± 1.5	41.4 ± 1.2	36.3 ± 1.5
0	0	0	0
MIC	1.9	7.8	15.6

In addition, Scopularide A and B showed a potent broad-spectrum anti-Helicobacter pylori activity with MIC values ranging from 7.81 to 15.63 µg/ ml (Table 1).

**Fig. 2:**
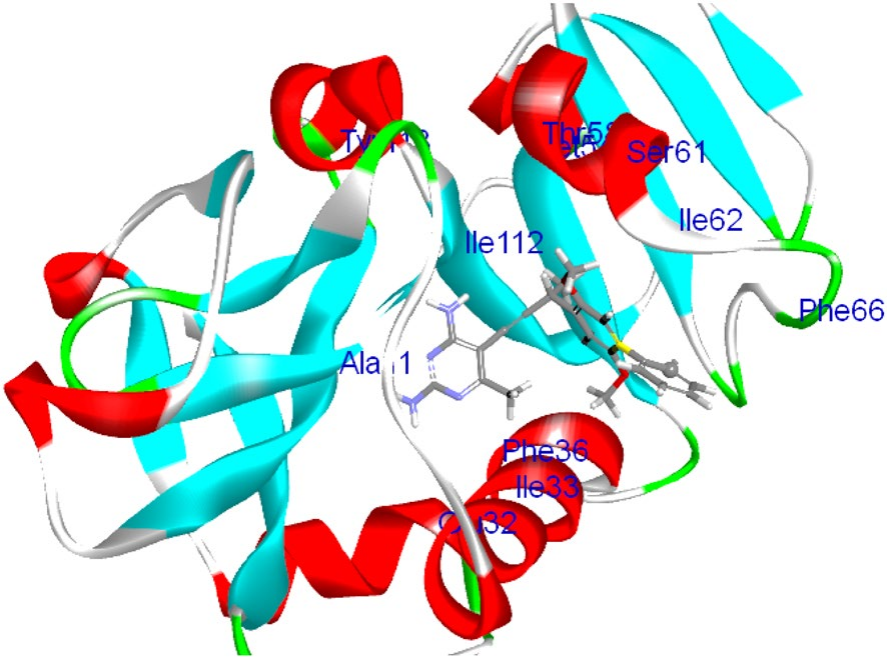
3D-interaction binding mode of the complexed ligand with *Candida albicans* dihydrofolate reductase

**Fig. 3.**
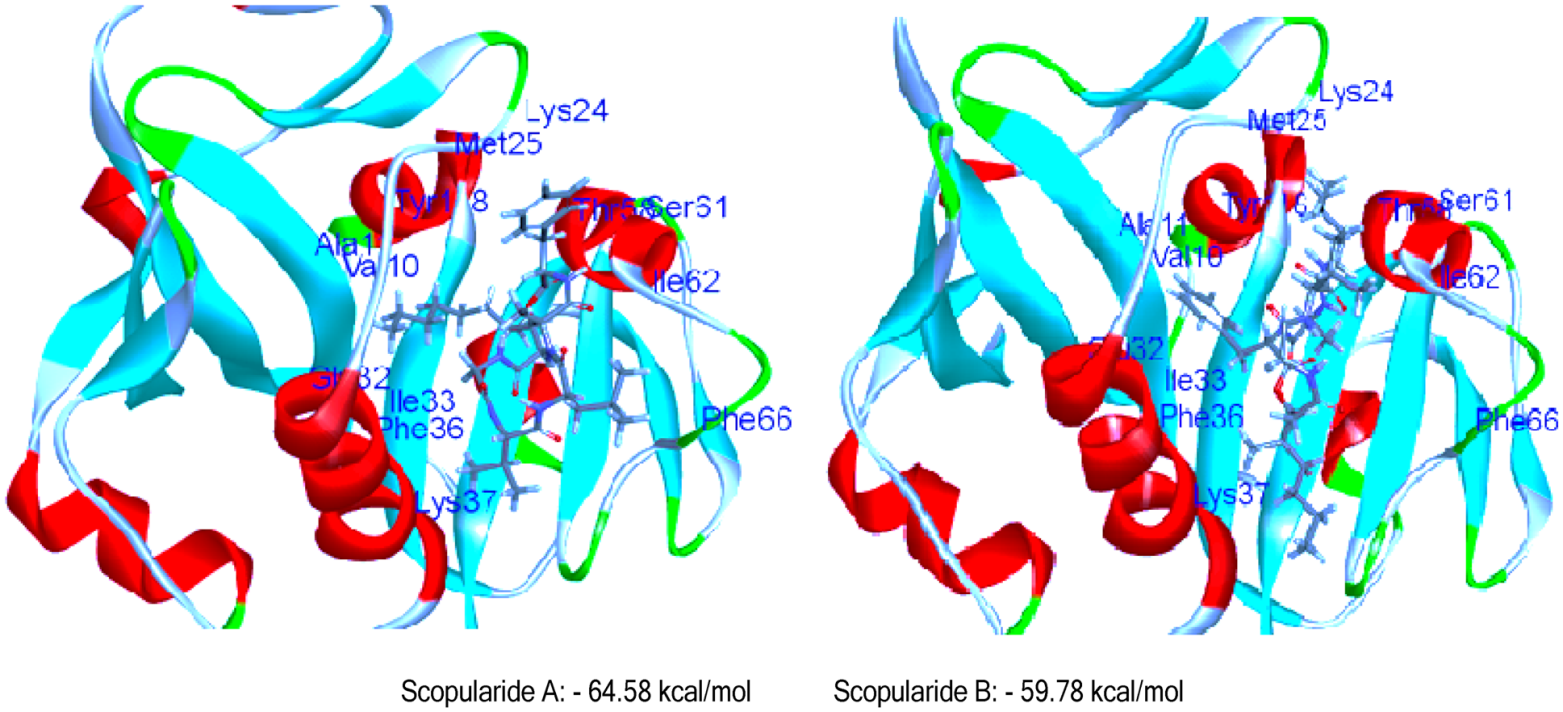
3D-interaction binding mode of Scopularide A and B with Candida albicans dihydrofolate reductase***.*** Scopularide A:− 64.58 kcal/mol Scopularide B:− 59.78 kcal/mol

Based on the results from the experimental testing, in silico investigations using docking analysis were conducted to explore the potential interaction of the isolated metabolites to get more insight into their mechanistic action against *Candida albicans* & *Aspergillus niger*. Their crystal structures complexed with appropriate ligands (5-[3-(2,5-dimethoxy-4-phenylphenyl)but-1-yn-1-yl]-6-methylpyrimidine-2,4-diamine and 2-acetamido-2-deoxy-beta-D-glucopyranose) were downloaded from protein data bank (PDB: 4HOE & 3K4P), respectively [28, 29]. Docking study was performed by configuring the binding mode of the bioactive conformer of the co-crystallized ligand for each type of fungi via C-Docker protocol in the Discovery studio 4.0 software program. The bioactive conformer of the *Candida albicans* ligand displayed C-DOCKER interaction energy of value -40.81 kcal/mol and was found to be embedded inside the binding site pocket and surrounded by key amino acids (Tyr 118, Ile 112, Phe 36, Glu 32 and Ala 11), as shown in **(**Fig. 2**).** By comparing the compounds with the previously mentioned ligand, the 6 docked compounds showed C-Docker interaction energies in the range of 64.58–10.17 kcal/mol with the highest interaction energies being displayed by the compounds scopularide A (pose 4) & scopularide B (pose 1), -64.58 and − 59.78 kcal/mol, respectively, and a comparable binding mode **(**Fig. 3**).**

**Fig. 4:**
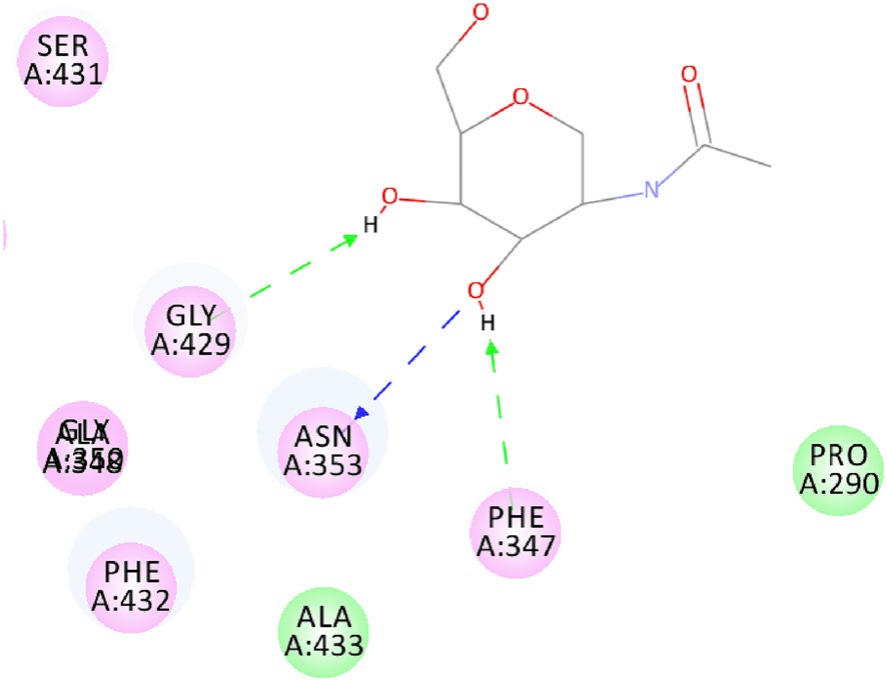
2D-interaction binding mode of a complexed ligand with *Aspergillus niger Phytase*

**Fig. 5:**
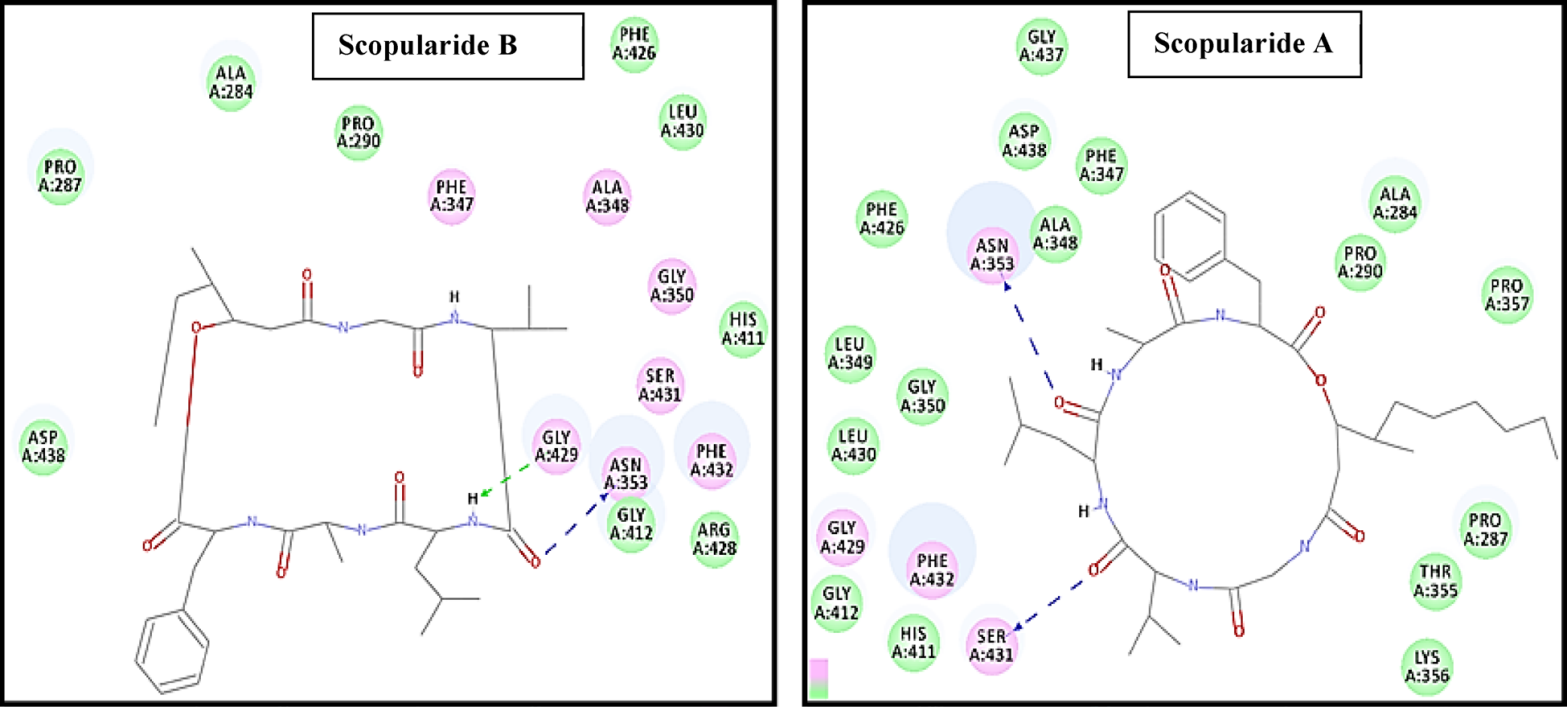
2D-Interaction binding mode of Scopularide** B** and **A** with *Aspergillus niger*

On the other hand, the bioactive conformer of the *Aspergillus niger* ligand presented three main hydrogen bonds with Gly 429, Asn 353 and Phe 347 as the main binding mechanism for hydrolase inhibition in *Aspergillus niger* **(**Fig. 4**)** and displayed C-DOCKER interaction energy of − 22.14 kcal/mol. The 6 docked compounds showed C-DOCKER interaction energies in the range of 51.00 to 13.07 kcal/mol and scopularide B (pose 5) and scopularide A (pose 2) were of highest interaction energies -51.00 and − 46.95 kcal/mol, respectively, and a comparable binding mode **(**Fig. 5**).** scopularide A can take a conformation which perfectly fits in the binding site of 4HOE & 3K4P due to the long hexyl chain. Hence, this bioactive conformation has a stable interaction energy, while scopularide B can occupy this perfect conformation and hence not as effective as scopularide A. Scopularide B displayed H-bonds with Gly 429 and Asn 353 while scopularide A displayed H-bonds with Asn 353 and Ser 431.

Green dotted lines represent the hydrogen bond acceptor and blue dotted lines represent hydrogen bond donor.

The parallel study of the mechanism of action of compounds on the bacteria; *Helicobacter pylori* was also performed using Discovery Studio 4.0 software program, where the crystal structures of the protein and its complexed ligand ((3R,4S)-1-[(4-amino-5H-pyrrolo[3,2-d]pyrimidin-7-yl)methyl]-4-{[4-hydroxybutyl)sulfanyl]methyl}pyrrolidin-3-ol) was downloaded from protein data bank under codes (PDB: 4WKO) [30]. The—[C-Docker interaction energy] of the bioactive conformer obtained for *H. pylori* complexed reference ligand was in the range of—(45.40–37.55) kcal/mol. s A & B were the most active and showed the best fit within the active site of *H-pylori* protein.

### Dynamic simulations

**Fig. 6:**
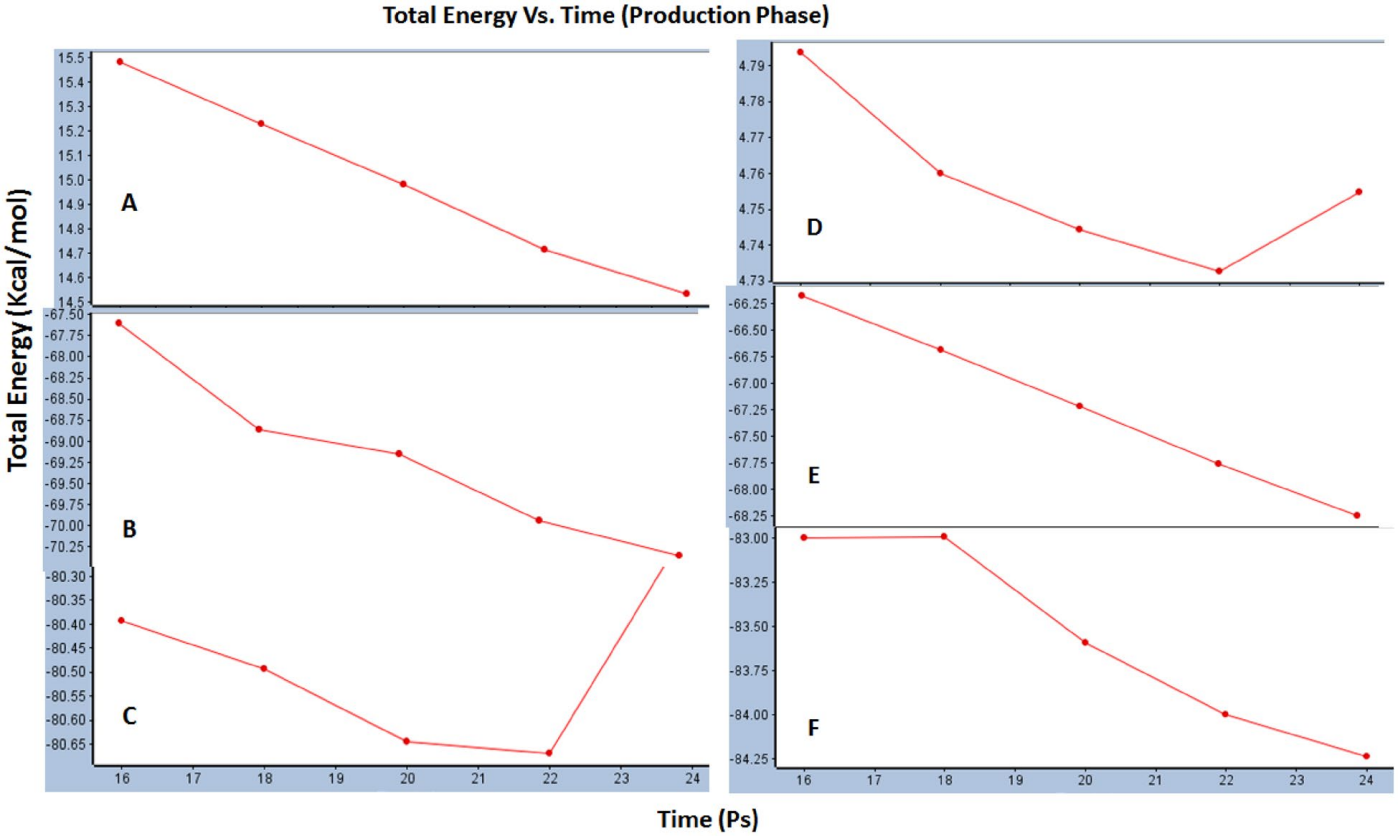
Total energy vs time obtained at production phase between **A** Reference ligand and DHFR, **B** Scopularide A and DHFR, **C** Scopularide B and DHFR, **D** Reference ligand and Phytase, **E** Scopularide A and Phytase, **F** Scopularide B and Phytase

Studies of dynamic simulation are very essential to figure out the stability of compounds during the interaction within the binding site of the target, hence to check the stability of the best two compounds; Scopularide A and B against both DHFR and Phytase enzymes, dynamic simulations were applied in their best-docked poses and compared to that of reference ligands for each enzyme. Five conformations were obtained for each compound th a mean total energy for the production phase in kcal/mol against time in Ps as shown in Fig. 6. Results revealed that for DHFR, the ligand stability increased overtime with a range of energy (14.5–15.5) kcal/mol while Scopularide A increased overtime with better stability of lower energy in the range of (− 67.5- − 70.25) kcal/mol. Meanwhile, Scopularide B increased to 22 Ps and then sharply decreased but within a better lower stable energy range (− 80.30- − 80.66) kcal/mol. New four hydrogen bonds were created between the reference ligand and DHFR, two between N1 and H30, H31 and two between N5 and H32, H33. As for Scopularide A, five hydrogen bonds have been recreated; N8–H73, N9–H77, N10–H78, N11–H79 and N12–H88, while Scopularide B displayed four hydrogen bonds; N8–H67, N9–H71, N10–H81, N12–H83 Additional file 1: Table S2.

Additionally, results of dynamic simulation studies with Phytase enzyme revealed that the ligand interaction stability increased overtime but decreased gradually after 22 Ps as shown in Fig. 6, while Scopularide A and B increased overtime with better total energy in ranges of (− 56.25 to − 58.25) and (− 83.00-− 84.25) kcal/mol respectively kcal/mol. On the other hand, ligand reformed new four hydrogen bonds with phytase; N9–H25, O10–H26, O11–H27 and O13–H28, while Scopularide A formed five hydrogen bonds; N8–H73, N9–H77, N10–H78, N11–H79 and N12–H88 and Scopularide B displayed also five new hydrogen bonds with phytase as follows; N8–H67, N9–H71, N10–H81, N11–H82 and N12–H83. From the above results, it can be concluded that Scopularide A and B could display stable interaction with both DHFR and phytase enzymes better than reference ligands and hence effective inhibition activity: Additional file 1: Table S4.

### Pharmacophore & IC_50_ prediction

**Fig. 7:**
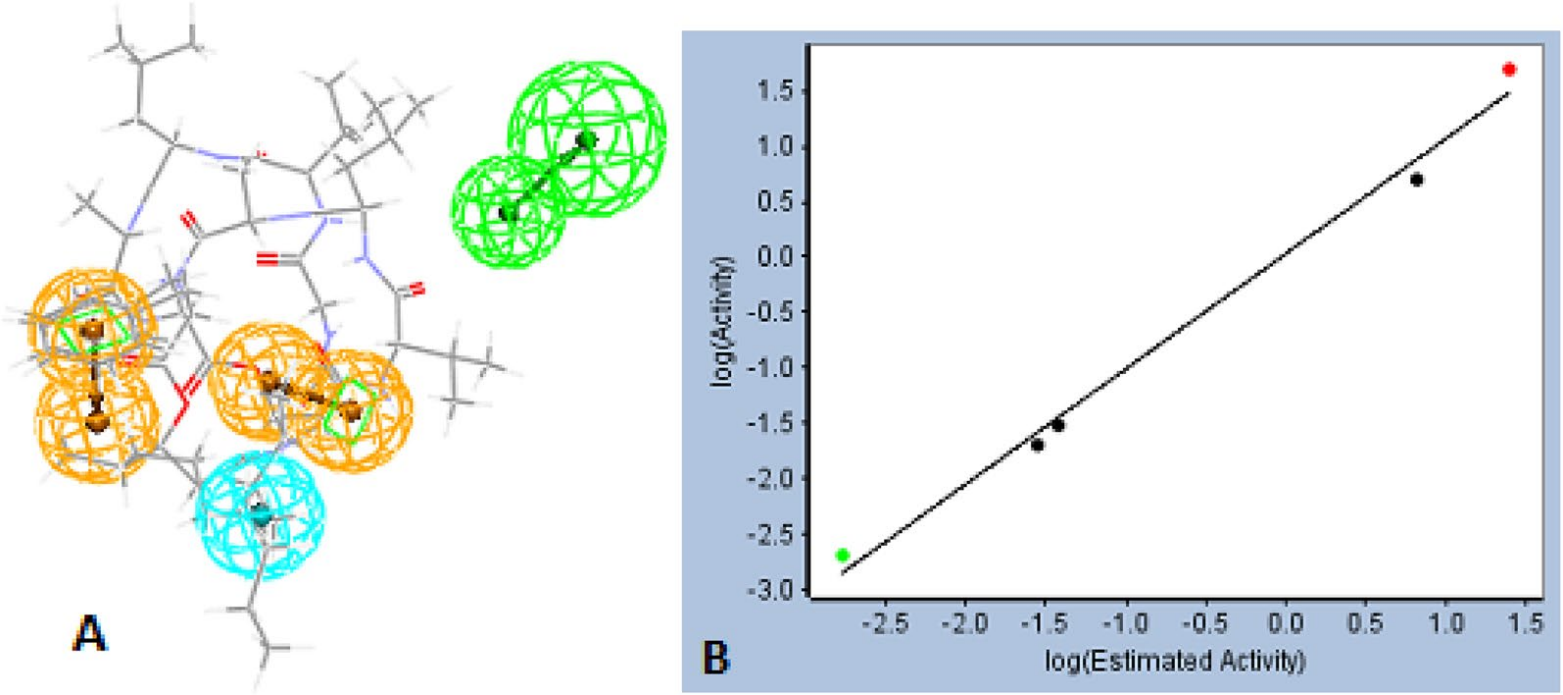
**A:** Pharmacophore model generated for structure-based targeting DHFR of *C. albicans*, Green color is indicated for Hydrogen Bond Acceptor (HBA), Cyan color is indicated for the hydrophobic binding site and Orange color is indicated for ring aromatic. **B:** A graphical display of the generated model for the near correlation between log estimated values and log actual values of IC_50_

To suggest the IC_50_ of the selected phytocompounds against DHFR enzyme, a pharmacophore study was performed where 7 compounds were selected from literature to generate a successful pharmacophore model out of 10 generated hypotheses. Detailed analysis of the generated hypotheses revealed a fixed cost of value: 32.0695 and a Null cost of value: 227.633, the best pharmacophore model selected based on total cost obtained to be near to fixed cost and far from null cost value and possessing the nearest correlation to 1. The first generated model was the most successful displaying total cost of value: 39.8973 and correlation factor = 0.995259. The obtained pharmacophore model as shown in Fig. 7 showed five essential structural features and eight different binding sites: two hydrogen bond acceptors (HBA), one hydrophobic pocket and four ring aromatic sites. This model showed estimated IC_50_ values of the training set of five compounds close enough to the reported ones as shown in Table 2 with acceptable fit value. Validation of the model was applied via the test set of two compounds which showed estimated IC_50_ comparable to those of actual ones with good fit value near 7. The phytocompounds were then screened using the selected pharmacophore model and showed expected IC_50_ in the range of (22.544—33.049) µM. Scopularide A and B displayed a mean of IC_50_ value of 24.7 µM. From these results, we can conclude that the phytocompounds could possess successful interaction against the DHFR of C. albicans with promising IC_50_ values.

**Table 2:** Results of pharmacophore model and screened compounds with their expected IC_50_ against *C. Albicans* DHFR:

Training set	Actual value (IC50) in µM	Estimate value	Fit value
65	0.03	0.0381054	8.80481
66	0.02	0.0282653	8.93455
Methotrexate	0.002	0.00165745	10.1664
Epigallocatechin-3-gallate	5	6.85022	6.5501
Trimethoprim	50	24.7068	5.99298
Test set	**Actual value (IC50) in µM**	**Estimate value**	**Fit value**
67	0.049	0.357903	7.83204
Pyrimethamine	4.8	15.7163	6.18945
Phytocompounds		**Estimate value**	**Fit value**
1		33.049	3.86664
2		24.729	5.99258
3		24.496	2.9967
4		22.544	6.03277
5		24.710	5.99291
6		24.705	5.99301

### Cytotoxic activity of the crude *A. flavus* extract

Upon the assessment of the fungal extract on MCF-7 cancer cell line, it was found that *Aspergillus flavus* showed a significant activity against the breast cancer cell line with IC_50_ 30.46 mg/ml.

## Discussion

Recent studies have demonstrated that endophytic microbes isolated from marine produce a variety of bioactive metabolites with different biological properties [31]. The genus *Sarcophyton* has been identified as a rich source of endophytic fungus [19, 32, 33] with beneficial bioactivities such as anticancer, antimicrobial, antiviral [32], larvicidal [19], and antimalarial [7, 34].

In this study, the soft coral marine *S*. *ehrenbergi* was selected as a source of promising marine fungi, since it has been previously confirmed that fungi derived from marine soft coral exhibited strong antimicrobial activity against many human pathogenic microorganisms [35], possibly arising from their ability to protect their host from outer threats [36]. Therefore, exploring new fungal strains can result in the development of novel and rare compounds with biotechnological and therapeutic applications.

The crude fungal extract was separated by extensive chromatography using silica gel, Sephadex LH-20 and HPLC to give compounds 1–6, including anhydro mevalonolactone, phomaligol A, ergosterol, scopularide A and B and finally ditryptophenaline. Anhydro mevalonolactone (AMVL) was separated for the first time from *Aspergillus flavus.* Wattanachaisaereekul et al. reported that AMVL has several applications in the biotechnological and medical industries and has been employed as a precursor in the manufacture of insect pest control chemicals [37]. Phomaligol A had previously been linked to the blackleg fungus *Leptosphaeria maculans* and marine-derived *Aspergillus flavus,* showing antibacterial action against *Staphylococcus aureus*, methicillin-resistant *S. aureus*, and multidrug-resistant *S. aureus* [38]. Moreover, phomaligol A and ergosterol showed moderate anti-trypanosomal activity against *Trypanosoma* *brucei*. Furthermore, ergosterol exhibited a strong inhibitory effect towards HepG2, B16, LLC, MDA-MB-231 and 4T1 cell lines [39]. Cyclodepsipeptides are comprised of a vast family that constitutes widely diverse peptide-related natural products that are mostly made of hydroxy and amino acids linked by amide and ester linkage. This class of natural products have a wide distribution in macro-organisms as well as microorganisms. On the other hand, cyclodepsipeptides have been associated to an array of diverse biological activities. Various activities were reported related to natural bioresources, and these diverse bioactivities were due to the presence of α-hydroxy acids which were like α-amino acids that occur readily in natural peptides. Moreover, due to frequent N-methylation of amino acid residues, which inhibits the action of enzymes and interferes with their bioavailability, they may also be able to withstand the action of certain hydrolyzing enzymes. Both cyclodepsipeptides, scopularide A and B isolated from *A*. *flavus* crude extract were subjected to various biological activities. In contrast to some reports [40], which documented that scopularide A and B exhibited no activity against Gram negative bacteria and moderate against gram positive bacteria, our results revealed that both Scopularide A and B possess significant broad-spectrum anti-*H*. *pylori* activity. Contrary to, Scopularide B revealed significant activity only against *Candida albicans*, while scopularide A showed significant antifungal activity against both *Candida albicans* and *A. niger.* As mentioned before, *Aspergillus* species are an important source of new bioactive metabolites with therapeutic values [41]. In this study, the crude extract of *A. flavus*, has been revealed to possess anticancer properties against human breast cancer cells.

## Conclusion

A total of six bioactive metabolites were identified from which, one was reported as novel to *Aspergillus flavus*-derived products. The compounds isolated and purified from an endophytic fungus *S. ehrenbergi* were classified as cyclodepsipeptides, pyrones and polyketides. In our study, Scopularides A and B revealed promising antifungal, and anti- *H. pylori activity.* Moreover, Scopularide A showed a potent activity against *A. niger*. Although further studies of these compounds on a wider range of clinical etiological agents of infection are needed to validate their capability as broad-spectrum antimicrobial, their antifungal activity was supported through molecular docking revealing that both scopularides A and B have good drug-like characteristics.

## Methods

### Fungal material and identification

The soft coral *Sarcophyton ehrenbergi* was collected in 2015 from Hurghada Red Sea, Egypt (10 meters depth), identified by Dr Hamada Ali of the National Institute of Oceanography and Fisheries, Hurghada, and a vouchered sample was kept at Ain Shams University under with code PHG-M-SE-259. The workup for the isolation and identification of the fungal strain was performed as previously reported [19].

### Cultivation, extraction, and isolation

Cultivation of the strain was performed as previously reported [19]. The crude extract (1.2 g) obtained was partitioned between n-hexane and 90% aqueous MeOH, giving 850 mg of 90% aqueous MeOH extract. The latter was subjected to vacuum liquid chromatography (VLC) on silica gel 60 eluting with gradient mobile phase (n-hexane-EtOAc 100:0 to 0:100, DCM-MeOH 100:0 to 0:100) to give eight fractions. Fraction 1 was purified using a semipreparative RP- HPLC column eluted with acetonitrile: water 9:1, in 90% gradient elution to yield ergosterol. Fraction 2 was applied on a silica gel column eluted with n-hexane: methylene chloride: MeOH yielding 2 subfractions, subfraction 1 was further purified on semi-preparative RP-HPLC column eluted with acetonitrile: water in 50–100% gradient to yield phomaligol A. Subfraction 2 was further purified on semi-preparative RP-HPLC column eluted with acetonitrile: water in 50–100% gradient to yield anhydro-mevalonolactone. Fraction 3 was purified using LH-20 using MeOH to yield scopularide A and B. Fraction 4 was purified using semipreparative RP- HPLC column eluted with acetonitrile: water 4:6, in 40% gradient elution to yield ditryptophenaline [42, 43].

### Nuclear magnetic resonance (NMR) spectrometry

All NMR experiments were performed on Bruker Ascend 400/R (Bruker^®^, AVANCE III HD, 400 MHz, Switzerland) spectrophotometer in the Center for Drug Discovery, Research and Development, Faculty of Pharmacy at Ain Shams University [19, 44–46].

### Computational methods

Three discrete molecular docking studies were carried out for the 6 separated compounds against two different types of fungi that include *Candida albicans* and *Aspergillus niger* and one bacteria *Helicobacter pylori.* The 3D structure of the 6 separated compounds was prepared using the Discovery Studio Version 4.0 software and the simulation was applied by using CHARMm as forcefield as described by [47, 48]. In addition, the partial charge was applied by Merck Molecular Force Field (MMFF94). However, conformational models have been generated automatically. Using the crystal structure of *Candida albicans* dihydrofolate reductase complexed with 5-[3-(2,5-dimethoxy-4-phenylphenyl)but-1-yn-1-yl]-6-methylpyrimidine-2,4-diamine (UCP111E) as an oxido-reductase inhibitor ligand, the mode of action of compounds’ against fungi was explored [28]. Likewise, the crystal structure of *Aspergillus niger* phytase complexed with 2-acetamido-2-deoxy-beta-D-glucopyranose as hydrolase inhibitor ligand [29] was downloaded from a protein data bank under codes PDB: 4HOE & 3K4P, respectively. Meanwhile, the study against bacteria included the crystal structure of *Helicobacter pylori* 5ʹ -methylthioadenosine/S-adenosyl homocysteine nucleosidase (MTAN) complexed with hydroxybutylthio-DADMe-Immucillin-A ((3R,4S)-1-[(4-amino-5H-pyrrolo[3,2-d]pyrimidin-7-yl)methyl]-4-pyrrolidin-3-ol) as hydrolase inhibitor ligand [49] downloaded from protein data bank under codes: (PDB: 4WKO).

Some protocols have been applied for both studies where proteins were cleaned and the missing hydrogens together with their side chains were added. Energy minimization according to DS protocol was applied. Then, the C-Docker-CHARMm-based technique was performed as previously reported [50]. First, the complex ligand for each protein was docked separately in the protein binding pocket. Further, the 6 compounds were docked to generate ten binding modes for each compound. The best binding mode was selected based on the least ranked [C-Docker interaction energy] obtained.

### Dynamic simulation studies

To assure the stability of the inhibition activity of the phytocompounds during enzyme interaction, dynamic simulation studies have been performed for the interaction between the most active two compounds; (Scopularide A, Scopularide B) and the dihydrofolate reductase enzyme of c. albicans (4HOE) and phytase enzyme of *A. Niger* (3K4P) compared to the reference compounds utilized during the docking studies for each enzyme. The best pose of docking was selected for each ligand to perform standard dynamic cascades of the heating phase, equilibration phase and production phase. Forcefield applied for each phase was selected as the steepest descent and conjugate gradient, while the starting temperature was 50 K to 300 K. The velocity applied is up to 2000 and the time allowed was set in the range of 1–4 Ps (picosecond). The production phase type is NVT based on volume and the implicit solvent model selected was GBSW (Generalized Born With a simple Switching). Five different conformations were obtained for each docked ligand and the output molecule was subjected to analyze trajectory protocol for RMSD (Root Mean Square Deviation) production compared to the first frame [51].

### Pharmacophore studies

To suggest the IC_50_ of the phytochemicals that could be obtained against DHFR of *C. albicans* type, in silico pharmacophore study was generated selecting 7 compounds from literature which possessed promising reported in vitro IC_50_ against DHFR of *C. albicans* [52, 53]. Five compounds; (two series of biphenyl diamino pyrimidine derivatives-65,66-, methotrexate, trimethoprim and epigallocatechin-3-gallate) were selected as the training set. two attributes were added active for their reported IC_50_ and uncertainty column for error factor with value 1.5. After ligands were prepared and forcefield CHARMm and MMFF were applied, pharmacophore generation was conducted by selecting five features of HBO (Hydrogen Bond Donor), HBA (Hydrogen Bond Acceptor), Hydrophobic, POS (Positive Ionizable) and Ring Aromatic. The generation was set fast and allowed for 10 hypotheses generation. The best hypothesis was selected based on the total cost nearest to the fixed cost obtained from the study and far from the null cost. Furtherly, to validate the selected generated pharmacophore model two compounds (a third series of biphenyl diamino pyrimidine derivative-67- and Pyrimethamine) were selected from the literature as a test set [52, 53] and screened for their activity. The obtained estimated IC_50_ values were comparable to those in the literature and a reasonable fit value was obtained. Hence, our six phytochemicals were prepared as ligands and CHARMm, and MMFF forcefield were applied and the compounds were screened for their activity using the previously selected pharmacophore model. The estimated values were obtained together with their fit values [54].

### Determination of the minimal inhibitory concentration (MIC)

The antibacterial activity of tested compounds against *Helicobacter pylori* (ATCC 43526), was determined by a micro-well dilution method. The inoculum of *Helicobacter pylori* was prepared, and the suspensions were adjusted to 106 CFU/ml. The compounds under investigation and the standard drug (Clarithromycin) were prepared in dimethyl sulfoxide (DMSO). Subsequent twofold dilutions (1000–0.03 µg) were performed in a 96-well plate. Each well of the microplate included 40 μl of the growth medium (Brain Heart Infusion (BHI)) plus 10 % fetal bovine serum (FBS), 10 μl of inoculum and 50 μl of the diluted compounds. Clarithromycin and DMSO were used as positive and negative controls, respectively. The plates were incubated at 37 °C for 3 days, in 5 % O_2_, 10 % CO_2_, and 85 % N_2_ atmosphere. After that, 40 μl of 3-[4,5-dimethylthiazole-2-yl]-2,5-diphenyltetrazolium bromide (MTT) at a final concentration 0.5 mg/ml freshly prepared in water was added to each well and incubated for 30 min. The change to purple color indicated that the bacteria were biologically active. The inhibition percentage was calculated using the given formula:

$$\% {\text{ inhibition}} = {{{\text{Abs Control}} - {\text{Abs Sample}} \times 100} \mathord{\left/ {\vphantom {{{\text{Abs Control}} - {\text{Abs Sample}} \times 100} {\text{Abs Control}}}} \right. \kern-0pt} {\text{Abs Control}}}$$

The concentration of tested samples required for 90 % of inhibition (MIC 90) was determined from the corresponding dose-response curves. The MIC was taken as the lowest concentration where no change of color of MTT was determined using an automatic ELISA microplate reader at 620 nm. The MIC values were done in triplicates [55, 56] and the standard deviation was calculated using Microsoft office excel 2010.

### Antifungal activity using XTT assay

Microorganisms: *Aspergillus niger* (ATCC 6275) and yeast: *Candida albicans* (ATCC 10231). Calorimetric broth micro-dilution method using [2,3-bis(2-methoxy-4-nitro-5-sulfo-phenyl)-2H-tetrazolium-5-carboxanilide]-reduction (XTT) assay [57] was adopted to determine the inhibitory percentages and the MICs of test samples. All fungal strains were cultured at 37 °C on Sabouraud dextrose agar (Oxoid, UK) for 3–5 days. Fifty microliters (50 µl) of adjusted microbial inoculum (5×103 CFU/ml of RPMI-1640 medium) was added to each well, and then the microtiter plates were incubated in the dark at 37 °C for 24 h to promote hyphal germination. The extracts were serially diluted in DMSO, and then 50 µl of each dilution at final concentrations of 1000–0.24 µg/ml were added to the wells. Amphotericin B was used as the standard antifungal. After incubation, 100 µl of freshly prepared XTT (Sigma-Aldrich) were added, and incubated again for 1 h at 37 °C. Calorimetric variation in the XTT assay was measured using a microtiter plate reader (BioTECK, USA) at 492 nm. The inhibition percentage was calculated using the formula:

$$\% \,{\text{in}}{\kern 1pt} \,{\text{Inhibition}}\,{\text{at}}\,15.63{{\mu {\text{g}}} \mathord{\left/ {\vphantom {{\mu {\text{g}}} {{\text{ml}}}}} \right. \kern-0pt} {{\text{ml}}}} = N\left[ {{{1 - \left( {({\text{Abs (f + s)}} - {\text{Abss}}} \right)} \mathord{\left/ {\vphantom {{1 - \left( {({\text{Abs (f + s)}} - {\text{Abss}}} \right)} { - ({\text{Absf}} - {\text{Absb)]}} \times 100}}} \right. \kern-0pt} { - ({\text{Absf}} - {\text{Absb)]}} \times 100}}} \right]$$

Ab (f+ s) is of wells containing fungi with extracts, Abss is of wells containing extracts alone, Absf is of wells containing fungi alone, and Absb is of wells containing media alone [58]. The MIC was specified as the extract concentration that produced a 100 % decrease in optical density compared with the control growth results.

### Cytotoxic assay by MTT assay

Cytotoxic activity was investigated as defined by [59].

## Supplementary Information

**Additional file1: Figure S1.** APT spectrum of Phomaligol A. **Figure S2.** ^1^H-NMR spectrum of phomaligol A. **Figure S3.** APT spectrum of ergosterol. **Figure S4.** ^1^H NMR spectrum of ergosterol. **Figure S5.** ^1^H NMR spectrum of anhydro-mevalonolactone. **Figure S6.** APT spectrum of anhydro-mevalonolactone. **Figure S7.** HMBC correlation of anhydro-mevalonolactone**. Figure S8.** Key COSY correlations spectrum of anhydro-mevalonolactone. **Figure S9.** ^1^H-NMR spectrum of Ditryptophenaline. **Figure S10.** APT spectrum of Ditryptophenaline. **Figure S11.** HMBC correlation of Ditryptophenaline. **Figure S12.** Key COSY correlation of Ditryptophenaline. **Figure S13.** ^1^H-NMR spectrum of Scopularide A. **Figure S14.** APT spectrum of Scopularide A. **Figure S15.** Key COSY correlation of Scopularide A. **Figure S16.** HMBC correlation of Scopularide A. **Figure S17.** ^1^H-NMR spectrum of Scopularide B. **Figure S18.** APT spectrum of Scopularide B. **Figure S19.** HMBC correlation of Scopularide B. **Figure S20.** RMSD of Ligand docked with DHFR of *C. albicans *(4HOE). **Figure S21.** RMSD of Scopularide A docked with DHFR of *C. albicans *(4HOE). **Figure S22.** RMSD of Scopularide B docked with DHFR of *C. albicans* (4HOE). **Figure S23.** RMSD of Ligand docked with Phytase of *A. niger* (3K4P) **Figure S24.** RMSD of Scopularide A docked with Phytase of A. niger (3K4P). **Figure S25.** RMSD of Scopularide B docked with Phytase of *A. niger* (3K4P) **Table S1.** Docking analysis data. **Table S2.** Energy table results of dynamic simulation studies of ligand, Scopularide A and B docked against DHFR of C. *albicans*. **Table S3.** Energy table results of dynamic simulation studies of ligand, Scopularide A and B docked against Phytase of *A. niger*. **Table S4.** Structures of compounds used in pharmacophore studies

## Data Availability

All data generated or analysed during this study are included in this published article (and its additional file information files).
