# Supplementary material for: Antimicrobial activities of metabolites isolated from endophytic Aspergillus flavus of Sarcophyton ehrenbergi supported by in-silico study and NMR spectroscopy
Source: Fungal Biol Biotechnol. 2023 Aug 2;10:16. doi: 10.1186/s40694-023-00161-2 (PMC10394880; doi:10.1186/s40694-023-00161-2)
Supplement: Supplementary file 1 — Additional file1: Figure S1. APT spectrum of Phomaligol A. Figure S2. 1H-NMR spectrum of phomaligol A. Figure S3. APT spectrum of ergosterol. Figure S4. 1H NMR spectrum of ergosterol. Figure S5. 1H NMR spectrum of anhydro-mevalonolactone. Figure S6. APT spectrum of anhydro-mevalonolactone. Figure S7. HMBC correlation of anhydro-mevalonolactone. Figure S8. Key COSY correlations spectrum of anhydro-mevalonolactone. Figure S9. 1H-NMR spectrum of Ditryptophenaline. Figure S10. APT spectrum of Ditryptophenaline. Figure S11. HMBC correlation of Ditryptophenaline. Figure S12. Key COSY correlation of Ditryptophenaline. Figure S13. 1H-NMR spectrum of Scopularide A. Figure S14. APT spectrum of Scopularide A. Figure S15. Key COSY correlation of Scopularide A. Figure S16. HMBC correlation of Scopularide A. Figure S17. 1H-NMR spectrum of Scopularide B. Figure S18. APT spectrum of Scopularide B. Figure S19. HMBC correlation of Scopularide B. Figure S20. RMSD of Ligand docked with DHFR of C. albicans (4HOE). Figure S21. RMSD of Scopularide A docked with DHFR of C. albicans (4HOE). Figure S22. RMSD of Scopularide B docked with DHFR of C. albicans (4HOE). Figure S23. RMSD of Ligand docked with Phytase of A. niger (3K4P) Figure S24. RMSD of Scopularide A docked with Phytase of A. niger (3K4P). Figure S25. RMSD of Scopularide B docked with Phytase of A. niger (3K4P) Table S1. Docking analysis data. Table S2. Energy table results of dynamic simulation studies of ligand, Scopularide A and B docked against DHFR of C. albicans. Table S3. Energy table results of dynamic simulation studies of ligand, Scopularide A and B docked against Phytase of A. niger. Table S4. Structures of compounds used in pharmacophore studies [file 40694_2023_161_MOESM1_ESM.docx]

**Additional file**

Article

**In silico profile, NMR-spectroscopy, and antimicrobial study of extracts from an endophytic *Aspergillus flavus* of *Sarcophyton ehrenbergi***


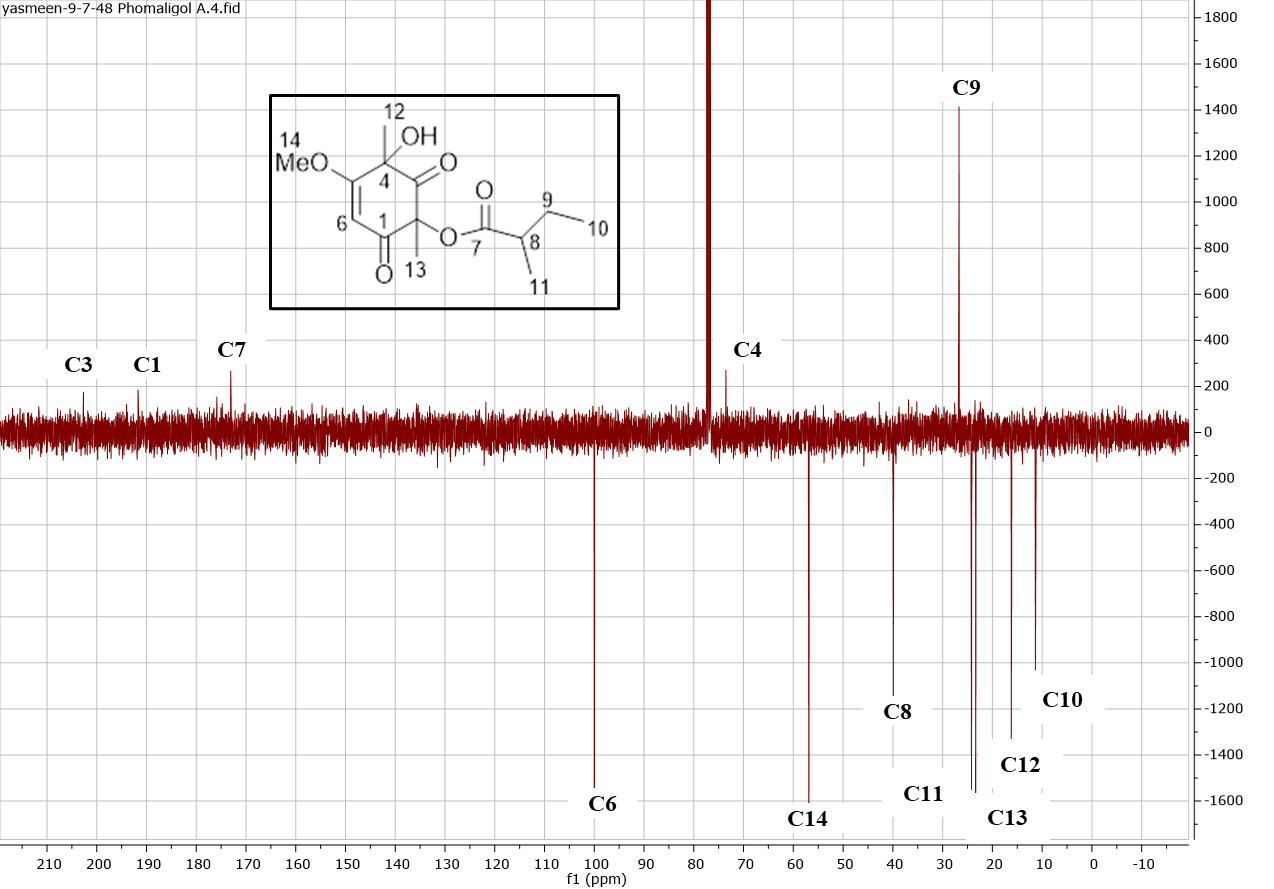


Figure S1. APT spectrum of Phomaligol A


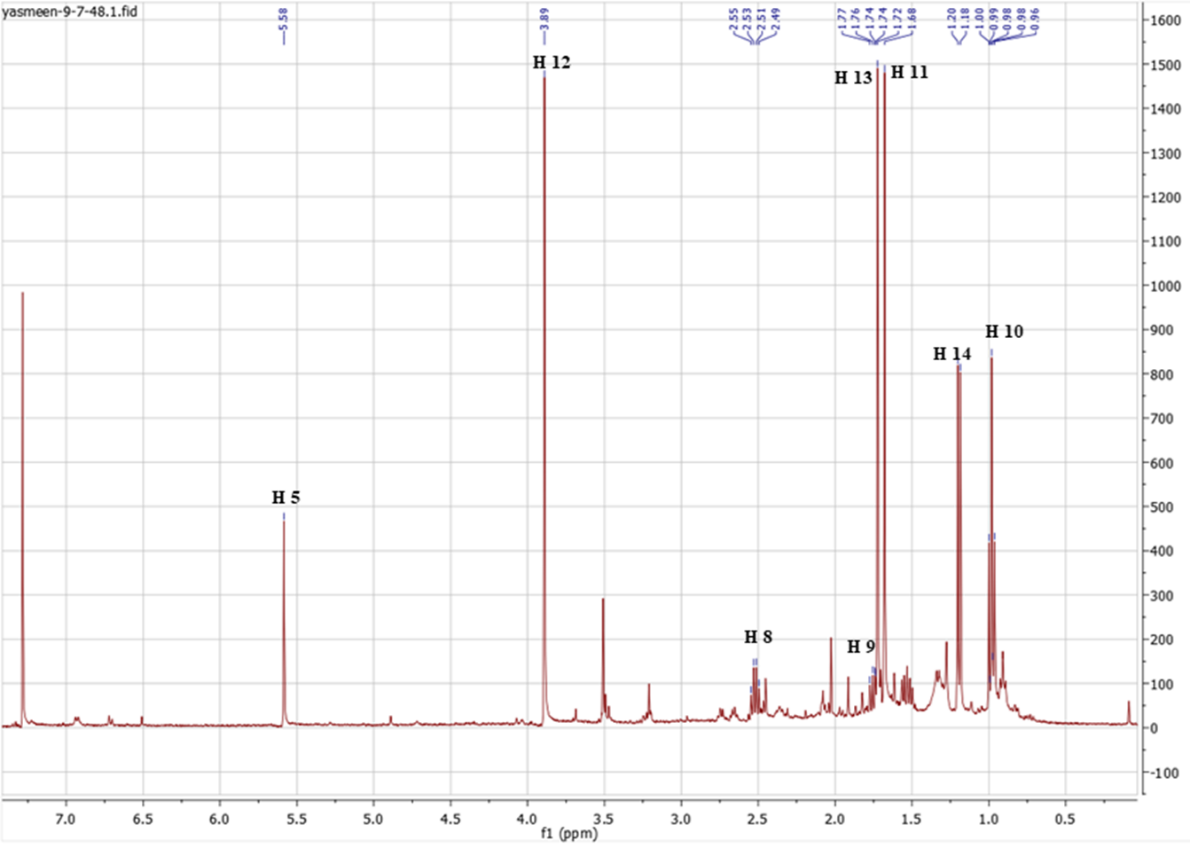


Figure S2. ^1^H-NMR spectrum of phomaligol A


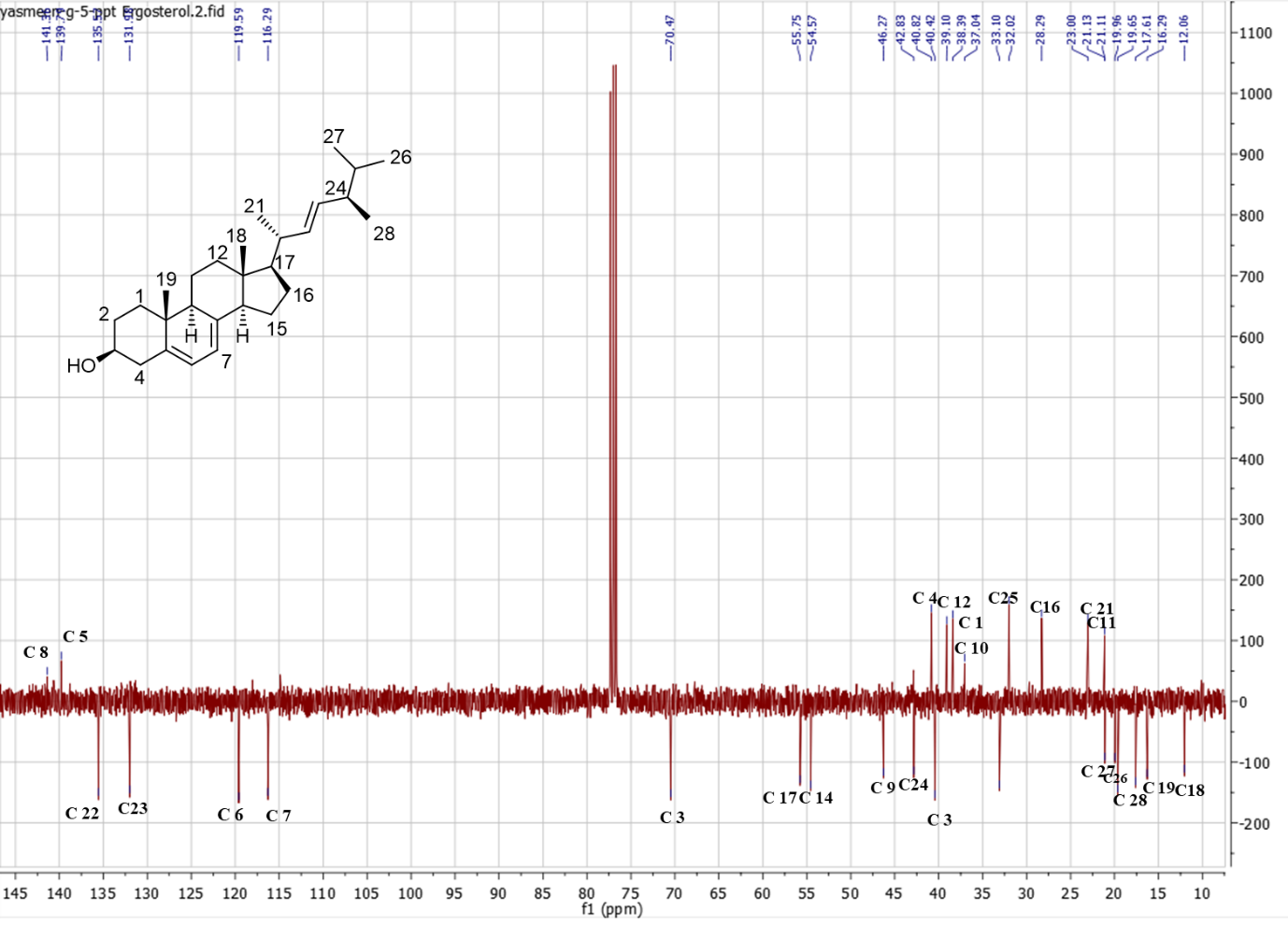


Figure S3. APT spectrum of ergosterol.

*
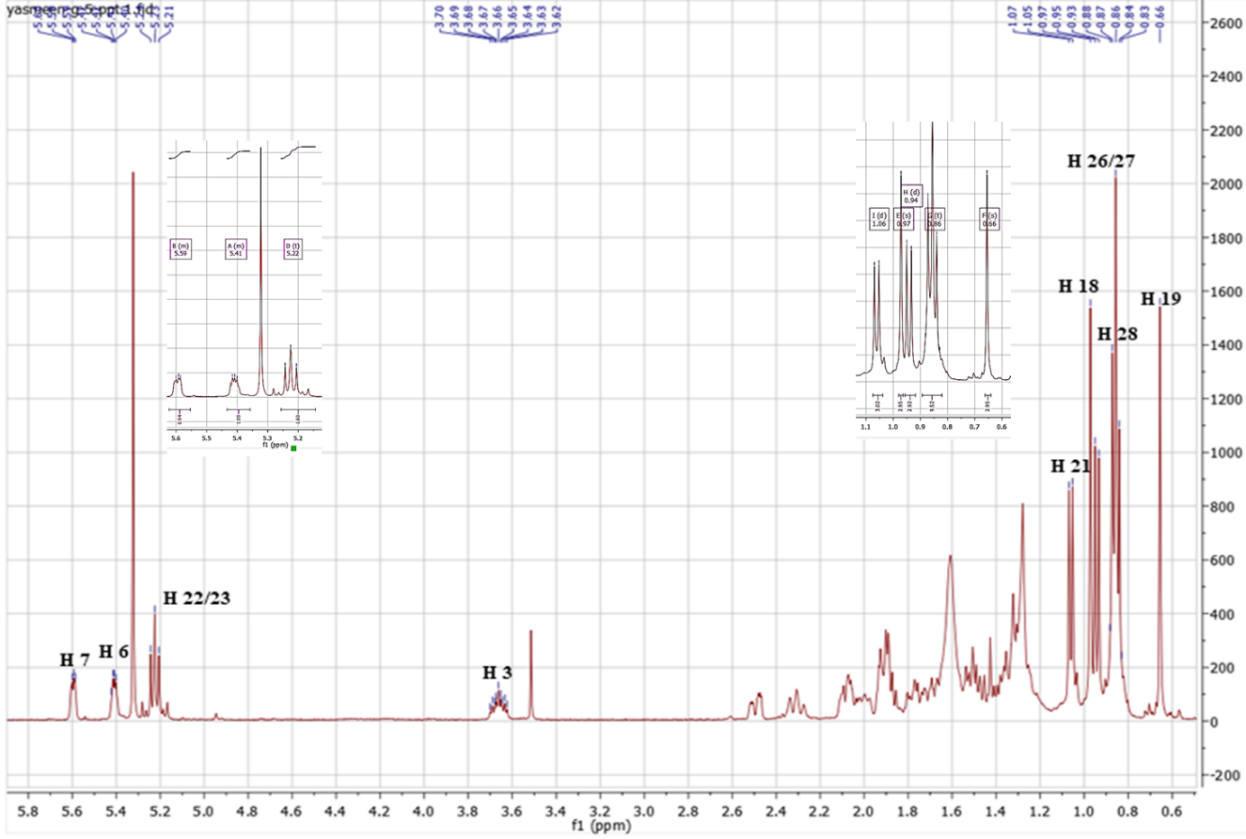
*

Figure S4. ^1^H NMR spectrum of ergosterol.


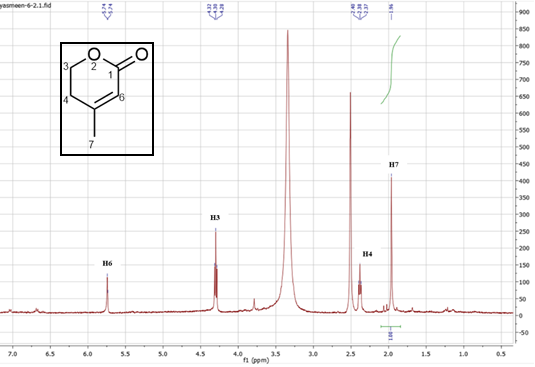


Figure S5. ^1^H NMR spectrum of anhydro-mevalonolactone.


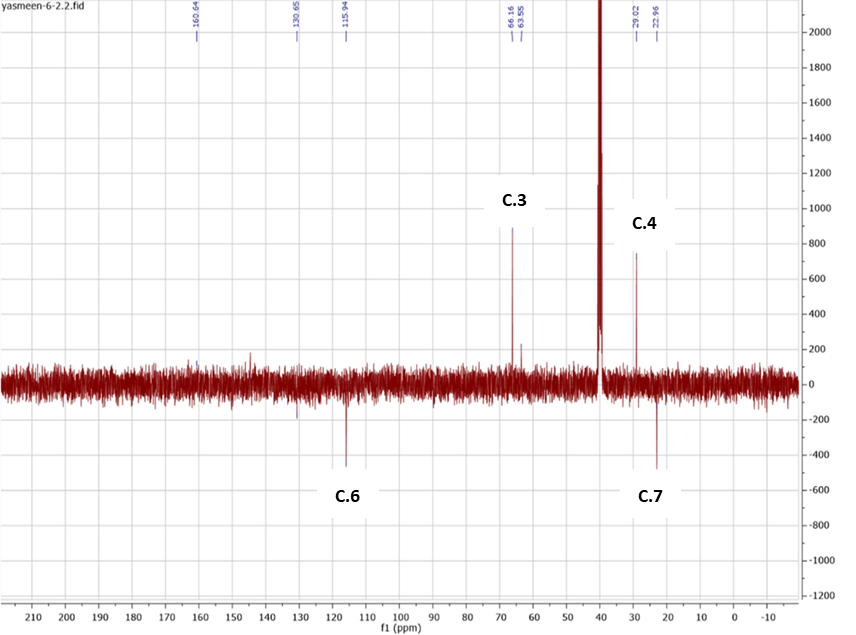


Figure S6. APT spectrum of anhydro-mevalonolactone.


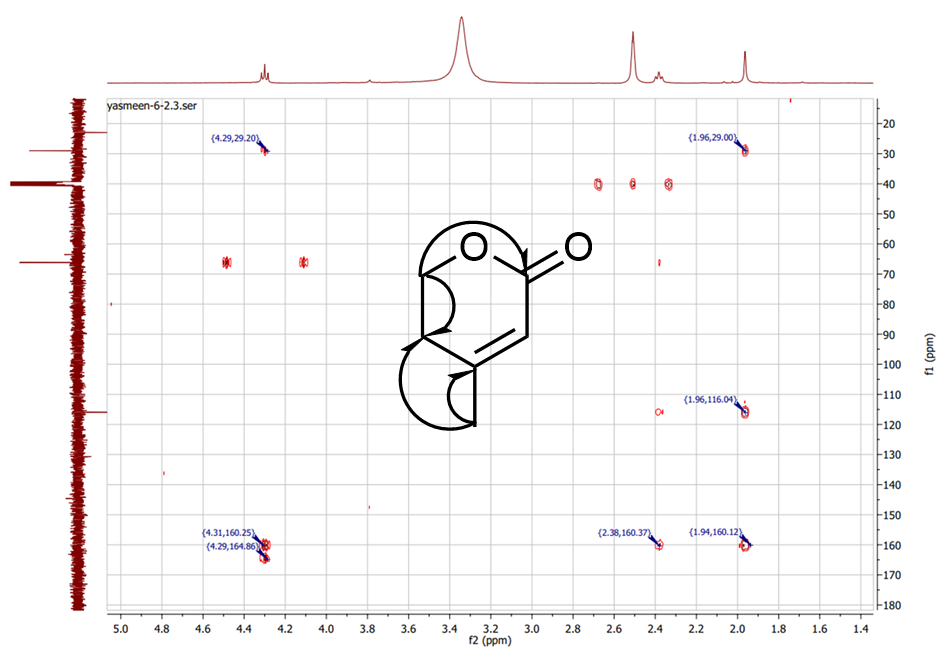


Figure S7. HMBC correlation of anhydro-mevalonolactone


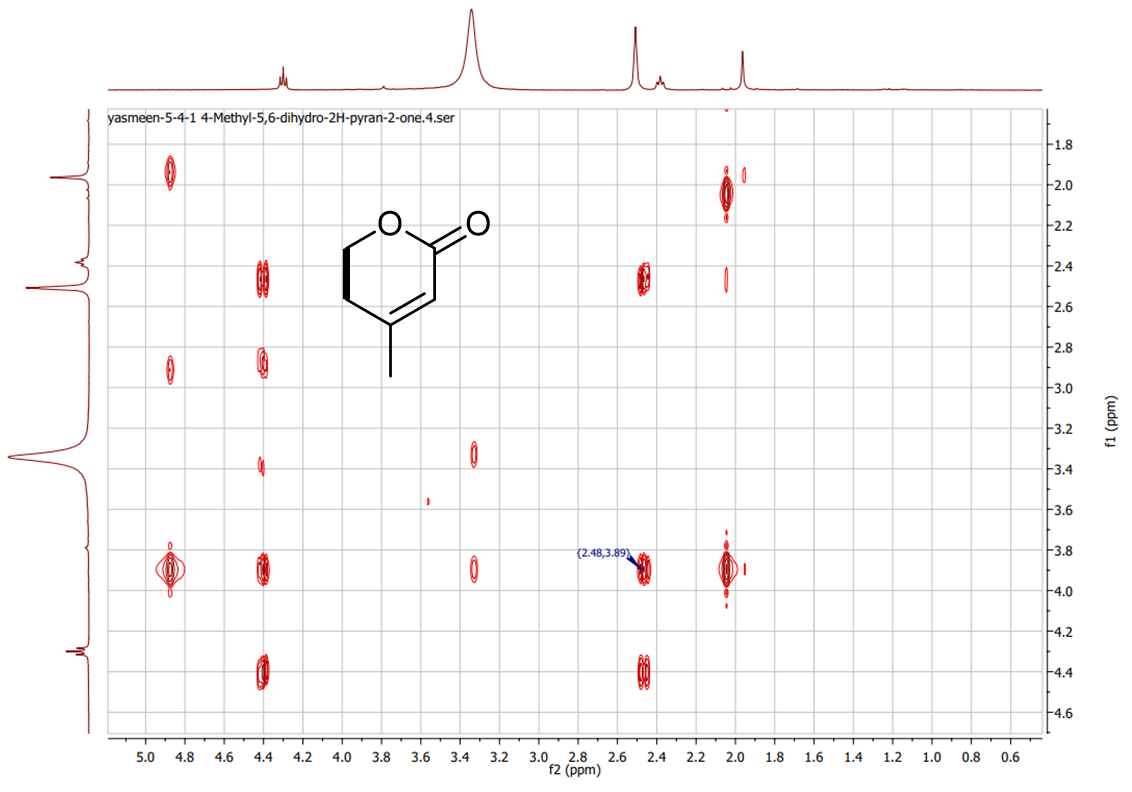
Figure S8. Key COSY correlations spectrum of anhydro-mevalonolactone.


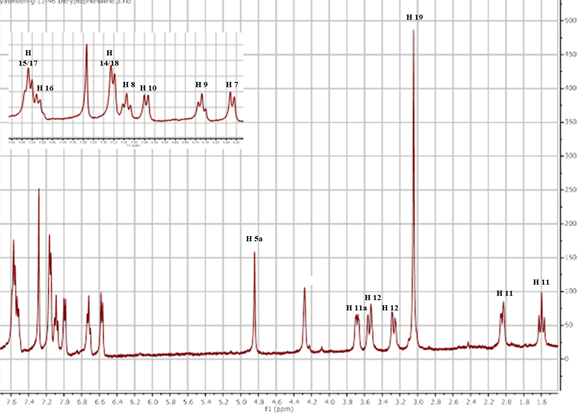


Figure S9. ^1^H-NMR spectrum of Ditryptophenaline


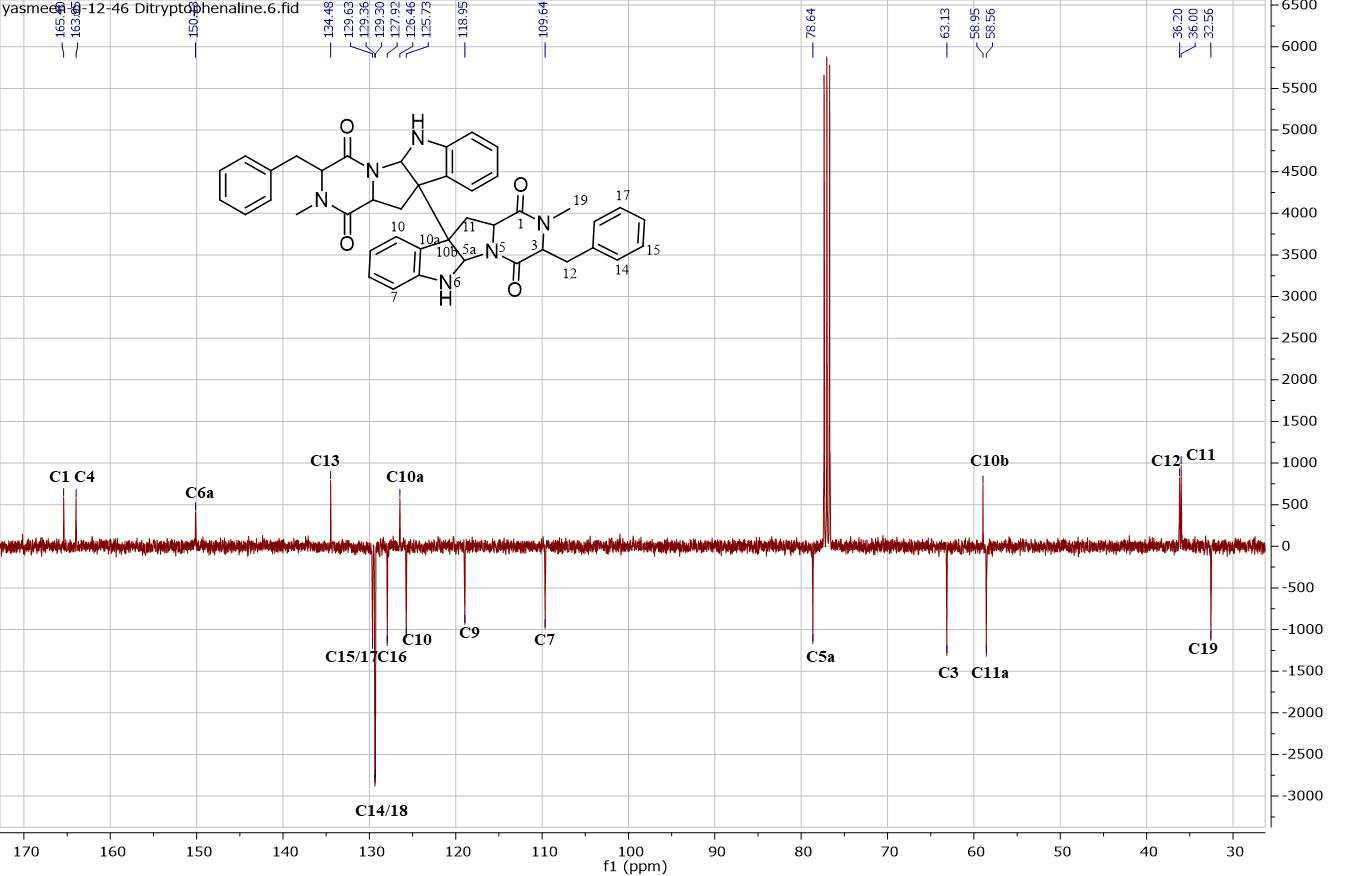


Figure S10. APT spectrum of Ditryptophenaline
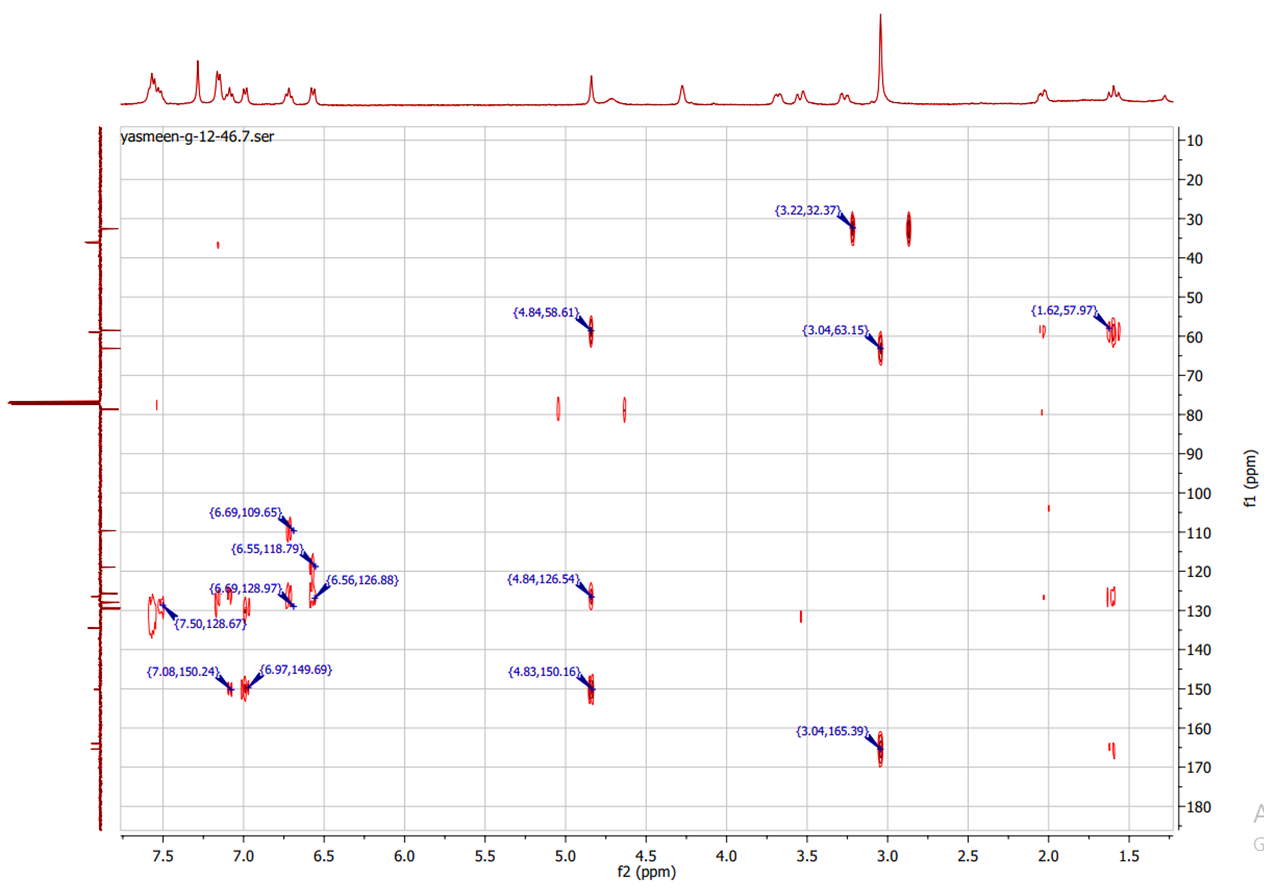


Figure S11. HMBC correlation of Ditryptophenaline


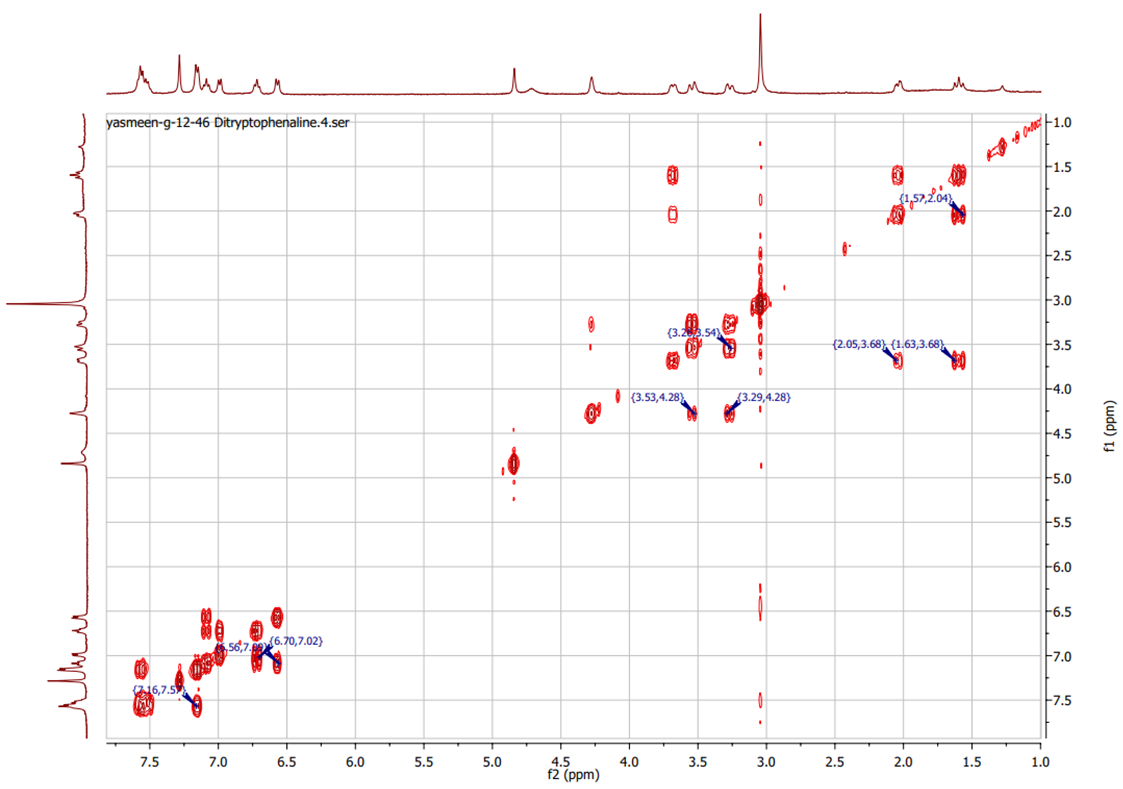


Figure S12. Key COSY correlation of Ditryptophenaline


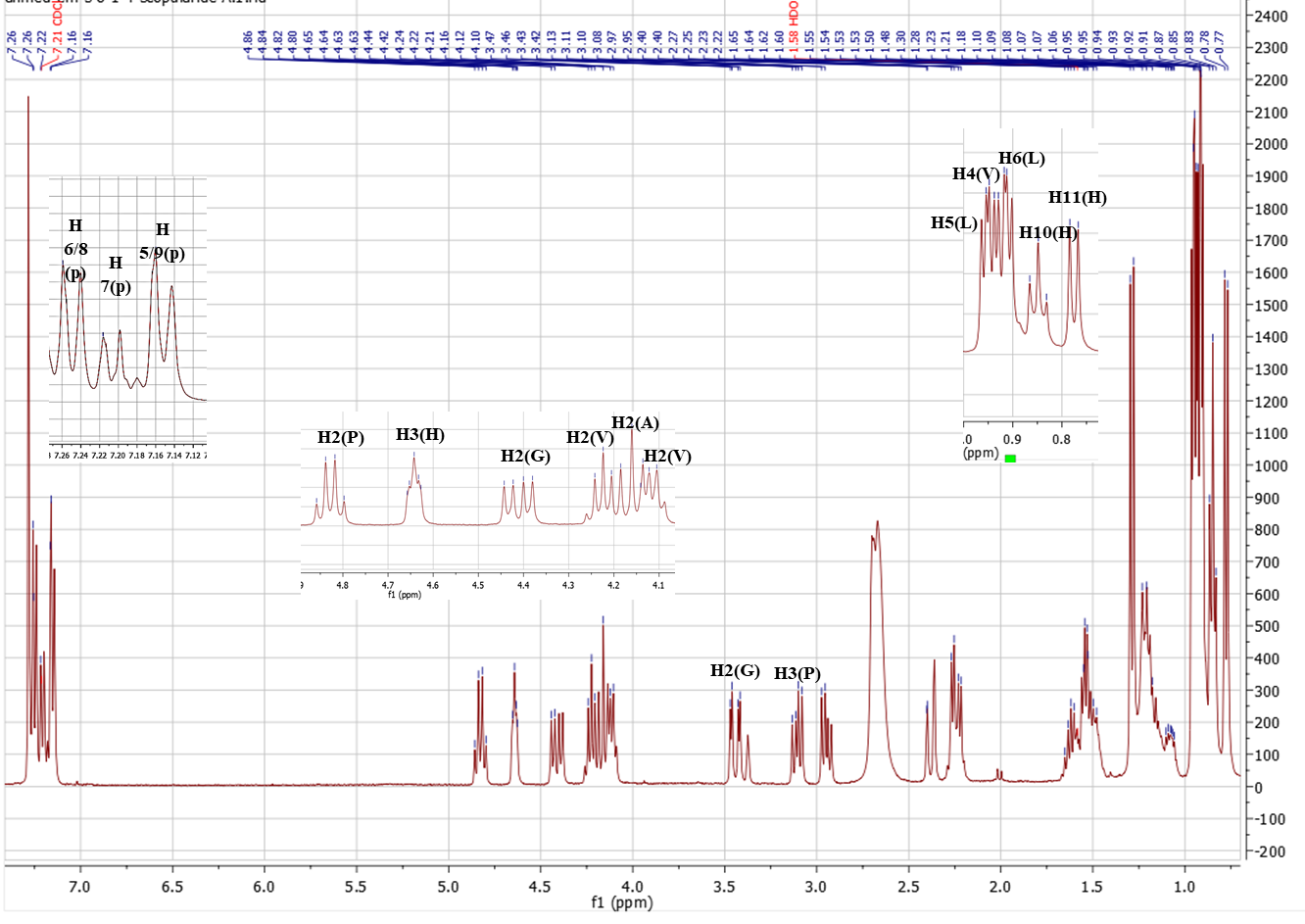


Figure S13. ^1^H-NMR spectrum of Scopularide A


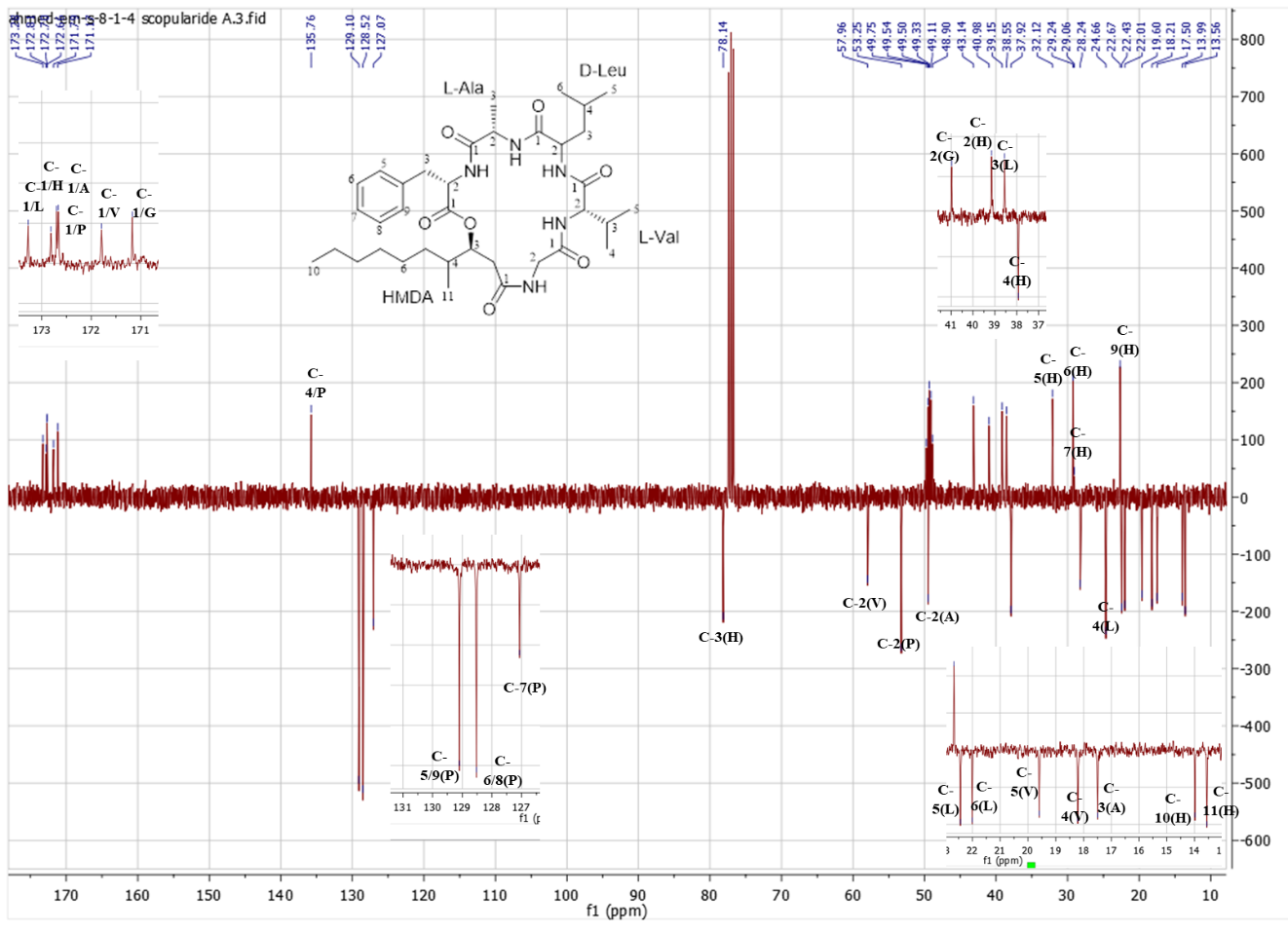


Figure S14. APT spectrum of Scopularide A


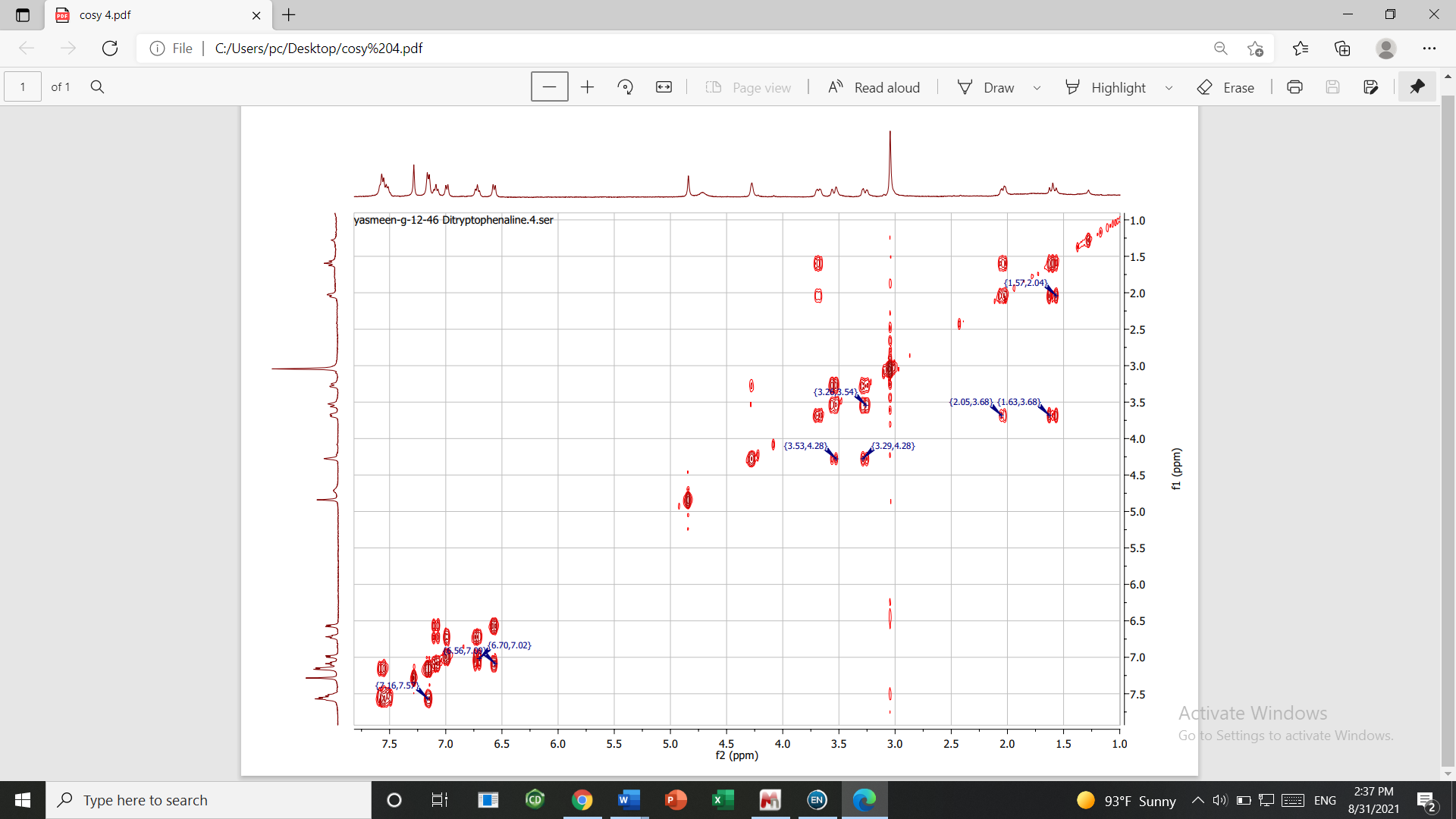


Figure S15. Key COSY correlation of Scopularide A


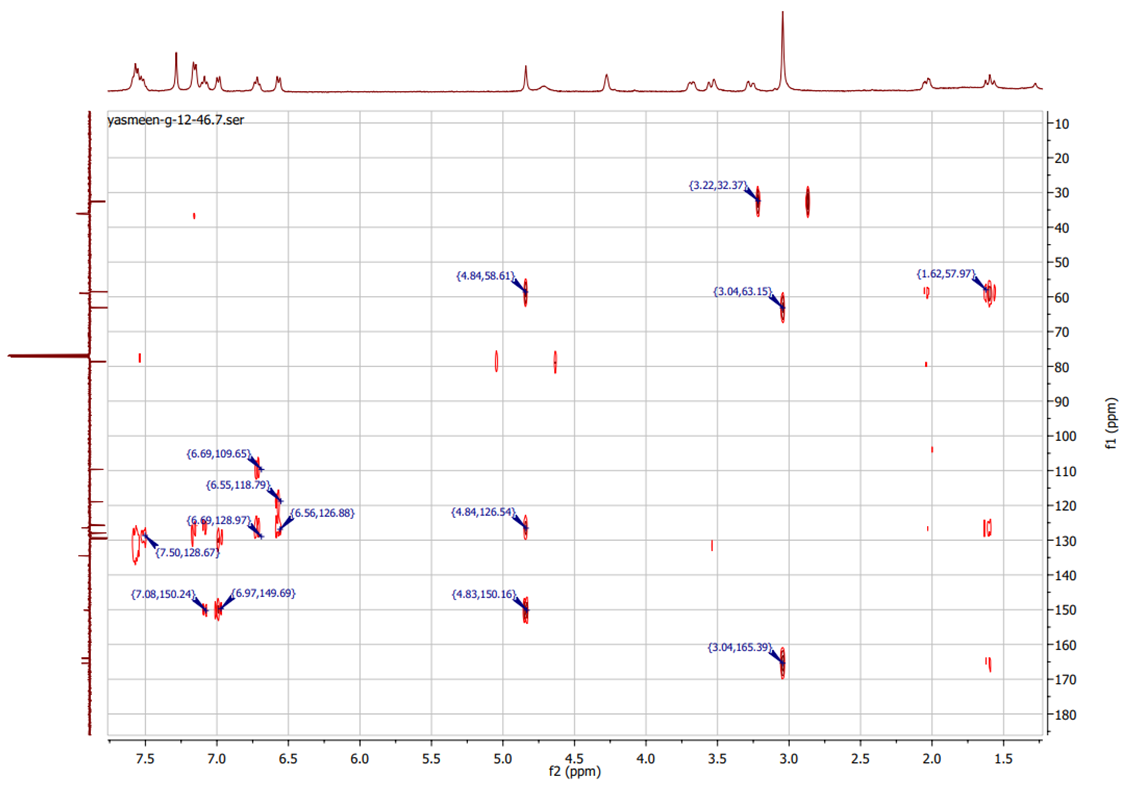


Figure S16. HMBC correlation of Scopularide A


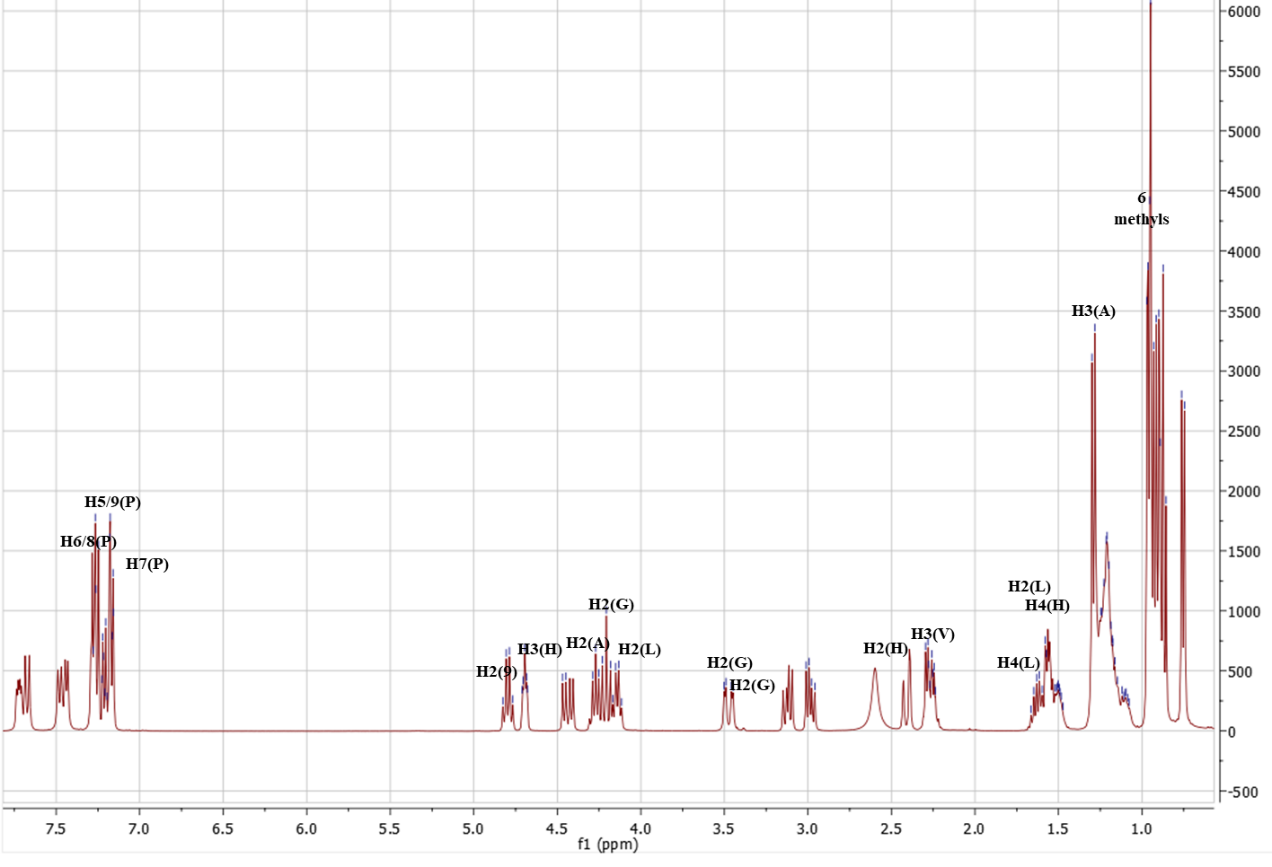


Figure S17. ^1^H-NMR spectrum of Scopularide B


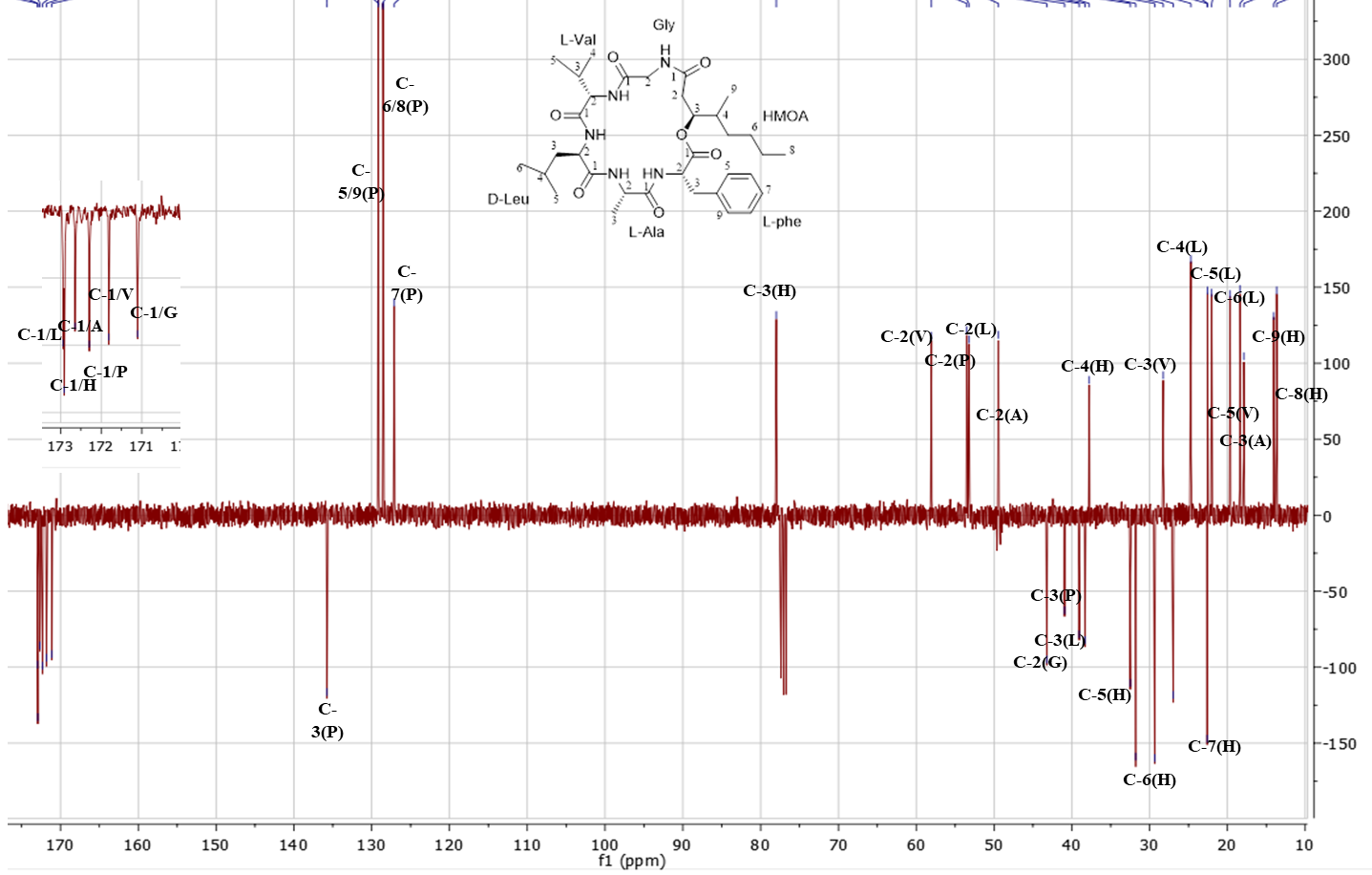


Figure S18. APT spectrum of Scopularide B


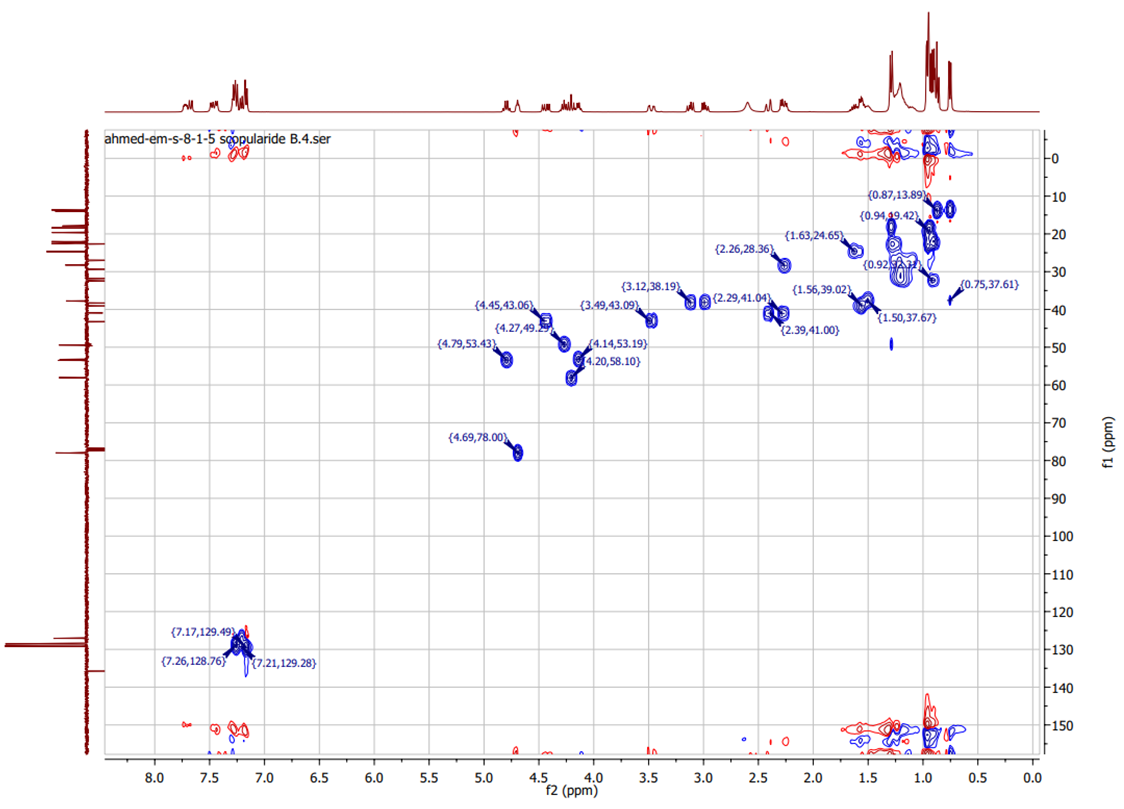


Figure S19. HMBC correlation of Scopularide B

Table S1: Docking analysis data.

| **Compound No.** | **Structure** | **-(C-Docker interaction energy) Kcal/mol.** | | |
| --- | --- | --- | --- | --- |
|  |  | **3K4P (*Aspergillus niger*)** | **4HOE (*Candida albicans*)** | **4WKO**  **(*H. pylori*)** |
|  | 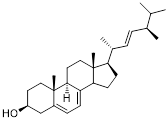 | 33.09 | 33.57 | 24.34 |
|  | 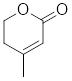 | 15.68 | 12.26 | 19.71 |
| 1. . | 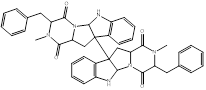 | 41.31 | 49.07 | 27.11 |
|  | 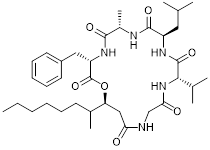 | 46.95 | 64.58 | 41.03 |
|  | 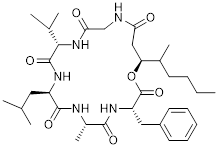 | 51.00 | 59.78 | 36.76 |

**Table S2: Energy table results of dynamic simulation studies of ligand, Scopularide A & B docked against DHFR of *C. albicans:***

| **The Name** | **Stage** | **Forcefield** | **Start Time (ps)** | **End Time (ps)** | **Initial Potential Energy (kcal/mol)** | **Total Energy (kcal/mol)** | **Potential Energy (kcal/mol)** | **Kinetic Energy (kcal/mol)** | **Temperature (K)** | **Van der Waals Energy (kcal/mol)** | **Electrostatic Energy (kcal/mol)** | **Initial RMS Gradient (kcal/(mol x A))** | **Final RMS Gradient (kcal/(mol x A))** |
| --- | --- | --- | --- | --- | --- | --- | --- | --- | --- | --- | --- | --- | --- |
| 4HOE | Minimization | CHARMm |  |  | -59.098 |  | -59.69 |  |  | 8.29 | -64.101 | 1.74 | 0.924 |
| 4HOE | Minimization2 | CHARMm |  |  | -59.69 |  | -61.744 |  |  | 7.916 | -63.247 | 0.924 | 0.097 |
| 4HOE | Heating | CHARMm | 0 | 4 | -61.744 | -47.97 | -55.864 | 7.894 | 61.589 | 8.436 | -60.879 | 1.303 | 10.159 |
| 4HOE | Equilibration | CHARMm | 4 | 14 | -55.864 | 15.936 | -25.564 | 41.5 | 323.781 | 9.219 | -63.877 | 10.159 | 22.593 |
| 4HOE | Production | CHARMm | 14 | 24 | -25.564 | 14.534 | -22.51 | 37.044 | 289.012 | 5.505 | -60.231 | 22.593 | 21.093 |
| Name | Stage | Forcefield | Start Time (ps) | End Time (ps) | Initial Potential Energy (kcal/mol) | Total Energy (kcal/mol) | Potential Energy (kcal/mol) | Kinetic Energy (kcal/mol) | Temperature (K) | Van der Waals Energy (kcal/mol) | Electrostatic Energy (kcal/mol) | Initial RMS Gradient (kcal/(mol x A)) | Final RMS Gradient (kcal/(mol x A)) |
| cpd_4 | Minimization | CHARMm |  |  | -183.267 |  | -184.577 |  |  | -23.434 | -159.624 | 1.942 | 0.56 |
| cpd_4 | Minimization2 | CHARMm |  |  | -184.577 |  | -210.535 |  |  | -28.243 | -179.681 | 0.56 | 0.094 |
| cpd_4 | Heating | CHARMm | 0 | 4 | -210.535 | -184.973 | -199.128 | 14.155 | 56.531 | -26.346 | -183.469 | 0.919 | 7.65 |
| cpd_4 | Equilibration | CHARMm | 4 | 14 | -199.128 | -66.364 | -148.087 | 81.723 | 326.387 | -23.428 | -177.187 | 7.65 | 17.214 |
| cpd_4 | Production | CHARMm | 14 | 24 | -148.087 | -70.354 | -142.279 | 71.925 | 287.254 | -22.356 | -166.367 | 17.214 | 15.033 |
| Name | Stage | Forcefield | Start Time (ps) | End Time (ps) | Initial Potential Energy (kcal/mol) | Total Energy (kcal/mol) | Potential Energy (kcal/mol) | Kinetic Energy (kcal/mol) | Temperature (K) | Van der Waals Energy (kcal/mol) | Electrostatic Energy (kcal/mol) | Initial RMS Gradient (kcal/(mol x A)) | Final RMS Gradient (kcal/(mol x A)) |
| cpd_5 | Minimization | CHARMm |  |  | -199.138 |  | -201.103 |  |  | -27.411 | -172.636 | 2.23 | 0.767 |
| cpd_5 | Minimization2 | CHARMm |  |  | -201.103 |  | -217.747 |  |  | -25.147 | -179.065 | 0.767 | 0.097 |
| cpd_5 | Heating | CHARMm | 0 | 4 | -217.747 | -193.516 | -207.048 | 13.532 | 57.224 | -23.614 | -179.452 | 1.016 | 7.577 |
| cpd_5 | Equilibration | CHARMm | 4 | 14 | -207.048 | -80.739 | -154.875 | 74.136 | 313.505 | -21.096 | -173.949 | 7.577 | 16.321 |
| cpd_5 | Production | CHARMm | 14 | 24 | -154.875 | -80.183 | -151.068 | 70.885 | 299.754 | -17.383 | -176.586 | 16.321 | 15.915 |

**Table S3: Energy table results of dynamic simulation studies of ligand, Scopularide A & B docked against Phytase of *A. niger:***

| **Name** | **Stage** | **Forcefield** | **Start Time (ps)** | **End Time (ps)** | **Initial Potential Energy (kcal/mol)** | **Total Energy (kcal/mol)** | **Potential Energy (kcal/mol)** | **Kinetic Energy (kcal/mol)** | **Temperature (K)** | **Van der Waals Energy (kcal/mol)** | **Electrostatic Energy (kcal/mol)** | **Initial RMS Gradient (kcal/(mol x A))** | **Final RMS Gradient (kcal/(mol x A))** |
| --- | --- | --- | --- | --- | --- | --- | --- | --- | --- | --- | --- | --- | --- |
| 3K4P | Minimization | CHARMm |  |  | -31.497 |  | -32.357 |  |  | -2.88 | -17.756 | 2.805 | 0.909 |
| 3K4P | Minimization2 | CHARMm |  |  | -32.357 |  | -34.588 |  |  | -2.55 | -22.983 | 0.909 | 0.082 |
| 3K4P | Heating | CHARMm | 0 | 4 | -34.588 | -27.832 | -32.057 | 4.225 | 64.427 | -2.875 | -23.956 | 5.26 | 8.283 |
| 3K4P | Equilibration | CHARMm | 4 | 14 | -32.057 | 4.864 | -13.403 | 18.267 | 278.552 | 0.355 | -22.039 | 8.283 | 20.44 |
| 3K4P | Production | CHARMm | 14 | 24 | -13.403 | 4.755 | -17.588 | 22.343 | 340.711 | -1.138 | -17.958 | 20.44 | 17.344 |
| Name | Stage | Forcefield | Start Time (ps) | End Time (ps) | Initial Potential Energy (kcal/mol) | Total Energy (kcal/mol) | Potential Energy (kcal/mol) | Kinetic Energy (kcal/mol) | Temperature (K) | Van der Waals Energy (kcal/mol) | Electrostatic Energy (kcal/mol) | Initial RMS Gradient (kcal/(mol x A)) | Final RMS Gradient (kcal/(mol x A)) |
| cpd_4 | Minimization | CHARMm |  |  | -210.418 |  | -211.834 |  |  | -26.417 | -166.529 | 1.844 | 0.515 |
| cpd_4 | Minimization2 | CHARMm |  |  | -211.834 |  | -217.636 |  |  | -24.956 | -171.924 | 0.515 | 0.097 |
| cpd_4 | Heating | CHARMm | 0 | 4 | -217.636 | -192.358 | -206.027 | 13.669 | 54.591 | -23.367 | -175.386 | 0.647 | 7.453 |
| cpd_4 | Equilibration | CHARMm | 4 | 14 | -206.027 | -65.743 | -147.081 | 81.338 | 324.851 | -21.653 | -169.9 | 7.453 | 17.686 |
| cpd_4 | Production | CHARMm | 14 | 24 | -147.081 | -68.249 | -141.853 | 73.604 | 293.963 | -17.917 | -175.503 | 17.686 | 18.143 |
| Name | Stage | Forcefield | Start Time (ps) | End Time (ps) | Initial Potential Energy (kcal/mol) | Total Energy (kcal/mol) | Potential Energy (kcal/mol) | Kinetic Energy (kcal/mol) | Temperature (K) | Van der Waals Energy (kcal/mol) | Electrostatic Energy (kcal/mol) | Initial RMS Gradient (kcal/(mol x A)) | Final RMS Gradient (kcal/(mol x A)) |
| cpd_5 | Minimization | CHARMm |  |  | -205.806 |  | -207.724 |  |  | -20.987 | -172.34 | 2.307 | 0.715 |
| cpd_5 | Minimization2 | CHARMm |  |  | -207.724 |  | -219.314 |  |  | -23.353 | -180.079 | 0.715 | 0.083 |
| cpd_5 | Heating | CHARMm | 0 | 4 | -219.314 | -199.379 | -212.821 | 13.442 | 56.843 | -25.167 | -181.496 | 0.795 | 8.174 |
| cpd_5 | Equilibration | CHARMm | 4 | 14 | -212.821 | -82.861 | -151.167 | 68.306 | 288.849 | -20.004 | -176.177 | 8.174 | 16.412 |
| cpd_5 | Production | CHARMm | 14 | 24 | -151.167 | -84.236 | -146.862 | 62.626 | 264.83 | -26.352 | -166.643 | 16.412 | 17.135 |

**Figure S20: RMSD of Ligand docked with DHFR of *C. albicans* (4HOE):**

**
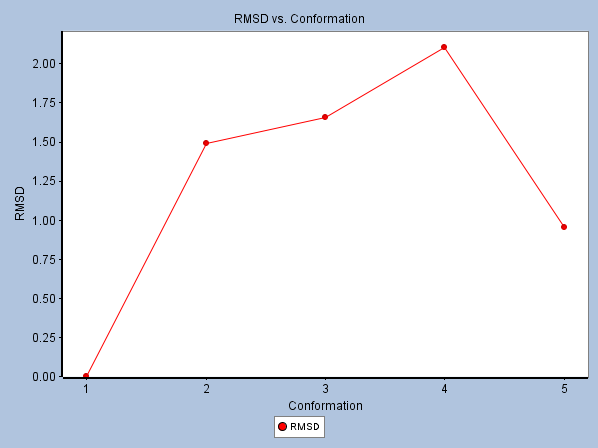
**

**Figure S21: RMSD of Scopularide A docked with DHFR of *C. albicans* (4HOE):**

**
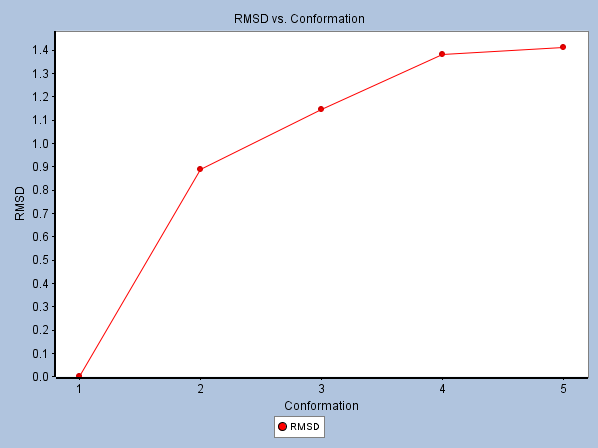
**

**Figure S 22: RMSD of Scopularide B docked with DHFR of *C. albicans* (4HOE):**

**
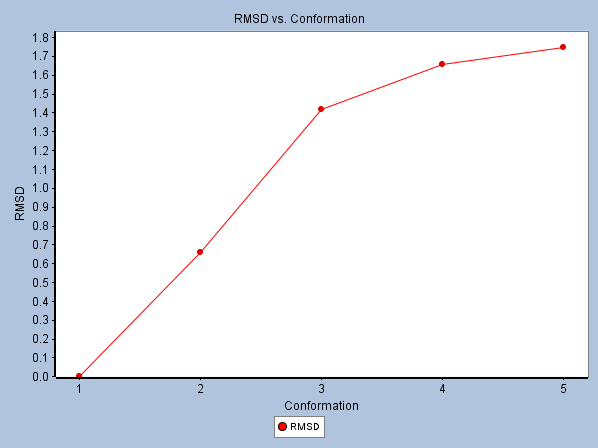
**

**Figure S23: RMSD of Ligand docked with Phytase of *A. niger* (3K4P):**

**
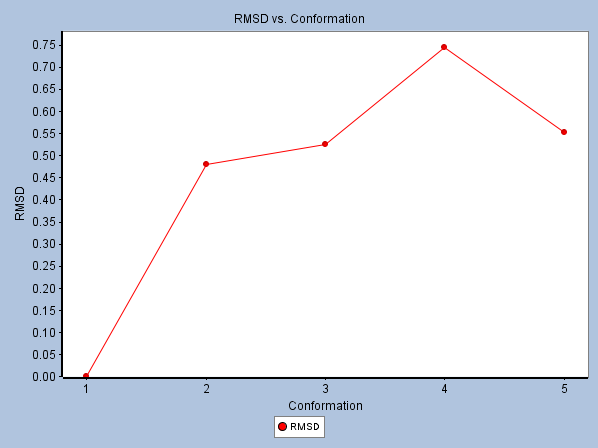
**

**Figure S24: RMSD of Scopularide A docked with Phytase of *A. niger* (3K4P):**

**
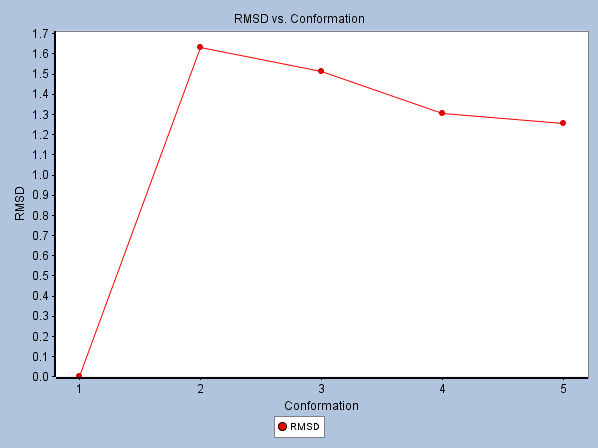
**

**Figure S25: RMSD of Scopularide B docked with Phytase of *A. niger* (3K4P):**

**
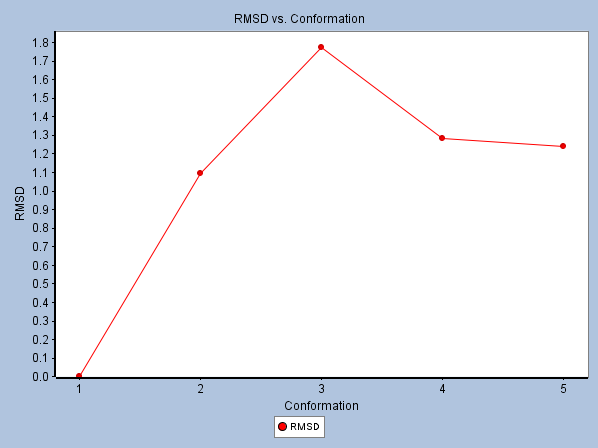
**

**S4: Structures of compounds used in pharmacophore studies:**

| **No.** | **Compound ID** | **Structure** |
| --- | --- | --- |
| **1** | **65** |  |
| **2** | **66** |  |
| **3** | **67** |  |
| **4** | **Methotrexate** |  |
| **5** | **Epigallocatechin-3-gallate** |  |
| **6** | **Pyrimethamine** |  |
| **7** | **Trimethoprim** |  |
